# Optimization of *Artemisia ordosica* crude polysaccharides on milk fatty acid composition in lactating donkeys and their effects on rectal microbiome and lactation performance

**DOI:** 10.3389/fmicb.2025.1682805

**Published:** 2025-11-04

**Authors:** Fanzhu Meng, Yanli Zhao, Yongmei Guo, Xiaoyu Guo, Qingyue Zhang, Shuyi Li, Yue Chi, Li Li, Fang Hui, Manman Tong, Sumei Yan

**Affiliations:** Inner Mongolia Key Laboratory of Animal Nutrition and Feed Science, College of Animal Science, Inner Mongolia Agricultural University, Hohhot, China

**Keywords:** *Artemisia ordosica* crude polysaccharides, lactation performance, fatty acid composition, rectal bacteria structure, lactating donkeys

## Abstract

**Introduction:**

This study evaluated the effects of dietary *Artemisia ordosica* crude polysaccharides (AOCP; 0.5 g/kg DM) supplementation on milk fatty acid profiles, rectal microbiota, enzymes related to lipid metabolism, and lactation performance in lactating Dezhou donkeys.

**Methods:**

A single-factor completely randomized design was used, with 14 lactating Dezhou donkeys (6.16 ± 0.67 years old, 250.06 ± 25.18 kg, parity 2.82 ± 0.48, 39.11 ± 7.42 days in lactation, each with a foal) randomly divided into two groups (*n* = 7/group). The CON group was fed a diet with a concentrate to forage ratio of 3:7, while the AOCP group received the same diet supplemented with 0.5 g/kg DM of AOCP. The trial lasted 10 weeks (including a 2-week adaptation period).

**Results and discussion:**

Compared with the CON group, AOCP supplementation significantly enhanced lactation performance (milk yield, fat, lactose) and the digestibility of DM, ADF, NDF, and elevated oleic acid, linoleic acid, eicosapentaenoic acid, as well as the unsaturated-to-saturated (U/S) and polyunsaturated-to-saturated (P/S) fatty acid ratios, while reducing saturated fatty acids and the c index. AOCP elevated acetate and butyrate in the rectum and the activity of enzymes related to lipid metabolism such as stearoyl-CoA desaturase, and increased the relative abundance of beneficial bacteria (*Eubacterium hallii group*, *Prevotella*, *Ruminococcus*), while decreasing potentially pathogenic bacteria *Streptococcus* and *norank_f_Lachnospiraceae*. In summary, AOCP may optimize the fatty acid composition of donkey milk and enhance lactation performance by modulating rectal bacteria structure, enzymes related to lipid metabolism, and nutrient utilization.

## 1 Introduction

Over the past two decades, donkey milk has attracted increasing attention as a functional food with potential beneficial effects on human health (Papademas et al., 2022). It has a low fat content and is rich in polyunsaturated fatty acids (PUFA) (Cimmino et al., 2023), making it especially suitable for people with cardiovascular diseases. Donkey milk contains various bioactive molecules with multifunctional properties. These include antimicrobial activity (e.g., lysozyme and lactoferrin), anti-inflammatory effects, and modulation of gut microbiota growth (Rubio, 2014; Yvon et al., 2018). Owing to its palatability and low levels of caseins and other allergenic proteins, it is particularly suitable for children with cow milk protein allergy (Mignone et al., 2015; Vincenzetti et al., 2017). However, donkey milk production remains limited, primarily owing to physiological constraints including a relatively small mammary gland, as well as an alveolar-dominant pattern of milk storage (Papademas et al., 2022). Nevertheless, lactation performance can be influenced by factors such as diet and animal health (Amirabadi Farahani et al., 2019). Therefore, improving milk production through nutritional strategies is of great importance.

*Artemisia ordosica* crude polysaccharide (AOCP) is extracted from *Artemisia ordosica*, a perennial dominant shrub species in the China-Mongolia region, and it contains diverse bioactive compounds, including polysaccharides, essential oils, flavonoids, and terpenoids (Zhou et al., 2019). These components exhibit broad-spectrum biological activities in livestock, such as antimicrobial, antioxidant, anti-stress, and anti-inflammatory effects, indicating significant application potential for AOCP in agriculture. Studies have shown that AOCP, as a feed additive, can enhance the antioxidant and anti-inflammatory capacities of lactating donkeys while improving the digestibility of nutrients (Li et al., 2024). Furthermore, dietary inclusion of *Artemisia montana* Pampan, a congeneric species of *Artemisia ordosica*, significantly elevates C18:3n3 and PUFA concentrations in beef cattle longissimus dorsi muscle, while concurrently reducing levels of C18:0, oleic acid (C18:1c9), and monounsaturated fatty acids (MUFA) (Kim et al., 2012). Intestinal microbiota play a critical role in monogastric animal physiology. A recent study by Guo et al. (2024) revealed that *Clostridium_sensu_stricto_1* in the rectum was significantly positively correlated with the digestibility of crude protein (CP), neutral detergent fiber (NDF), and acid detergent fiber (ADF) in late-gestation donkeys. Additionally, the *[Eubacterium]_coprostanoligenes_group* showed a strong positive correlation with dry matter (DM) digestibility. In rodent models, AOCP has been shown to significantly increase the relative abundance of *Bacteroidetes*, *unclassified_f_Lachnospiracea*e, and *Ruminococcus* (Xing et al., 2020), as well as promote the growth of probiotics including *Lactobacillus*, *Bifidobacterium*, and *Blautia* (Zeng et al., 2022). Nevertheless, studies investigating the effects of AOCP on fatty acid (FA) composition, lactation performance, and rectal bacteria structure in lactating donkeys remain scarce.

In our study, we hypothesized that AOCP supplementation modulates milk FA profiles and lactation performance via microbiota-mediated pathways. This study aims to provide a theoretical basis for the comprehensive development and utilization of *Artemisia ordosica* as a feed resource and establish a scientific foundation for improving donkey milk yield and quality.

## 2 Materials and methods

### 2.1 Animal ethics statement

The experiment was conducted at the demonstration base of Inner Mongolia Agricultural University, Inner Mongolia Grassland Yulv Science and Technology Animal Husbandry Co., Ltd. (Hohhot, China). The Animal Ethics and Welfare Committee approved the experimental procedures at the Inner Mongolia Agricultural University (NND2022050), which were under the university’s guidelines for animal research.

### 2.2 Preparation of *Artemisia ordosica* crude polysaccharides

The *Artemisia ordosica* (aerial parts) used in this study was collected during its July–August flowering period from sandy grasslands in Ordos, Inner Mongolia Autonomous Region. AOCP was prepared according to the method described by Xing et al. (2020). AOCP was extracted from the whole plant (excluding roots) using a water-extraction and ethanol-precipitation method. The dried plant material was ground into powder, defatted with petroleum ether, and then extracted with 15 volumes of distilled water at 60 °C for 4 h. The aqueous extract was filtered and concentrated. Polysaccharides were precipitated by adding anhydrous ethanol (4:1, v/v) to the concentrated supernatant and incubating at 4 °C for 48 h. The precipitate was collected by centrifugation at 12,000×*g* for 15 min and washed sequentially with petroleum ether, acetone, and ethanol. The resulting precipitate was redissolved in distilled water and deproteinized using the Sevag method and dialyzed. Finally, the product was freeze-dried to obtain AOCP powder, which was stored at −20 °C until use. The extracted AOCP yield was 55.6 g AOCP per kilogram of raw *Artemisia ordosica* material. Compositional analysis revealed that the major constituents of AOCP were polysaccharides (52.65%), proteins (2.39%), uronic acids (4.27%), and phenols (0.11%).

### 2.3 Animals, diets and experimental design

In a single factor completely randomized experimental design, a total of 14 lactating Dezhou donkeys (39.11 ± 7.42 days in milk, 2.82 ± 0.48 parity, 250.06 ± 25.18 kg of live weight) were randomly assigned to two groups, including the control (CON) and AOCP groups (*n* = 7). Based on the basal diet, the AOCP addition dose was 0 and 0.5 g/kg DM in the CON and AOCP groups, respectively. Table 1 shows the feed ingredients and nutrient composition (with a concentration-to-forage ratio of 3:7). The supplemented dose of AOCP was based on our previous study (Li et al., 2017). The experiment period lasted for 10 weeks, and included 2 weeks for adaptation (pretrial period) and 8 weeks for data and sample collection (experimental period).

**Table 1:** Composition and nutrient levels of basal diet (air-dry basis).

Items	Content
Ingredients (% of DM)	
Millet straw	33.97
Alfalfa	23.55
Corn	15.19
Soybean meal	8.55
Corn silage	12.49
DDGS (High fat)	1.80
Corn germ meal	1.80
Bran	0.90
NaCl	0.39
CaCO_3_	0.21
CaHPO_4_	0.66
Premix^1^	0.50
Total	100.00
DE (MJ/kg)^2^	13.82
DM	87.21
CP	12.28
EE	2.12
NDF	47.87
ADF	28.30
Ca	1.12
P	0.36

DE, digestible energy; DM, dry matter; CP, crude protein; EE, ether extract; NDF, neutral detergent fiber; ADF, acid detergent fiber; Ca, Calcium; P, Phosphorus.

1 Premix provided the following per kg of diet: vitamin A 6,000 IU, vitamin D 1,250 IU, vitamin E 15 IU, Fe 40 mg, Cu 8 mg, Zn 60 mg, Mn 60 mg, I 0.36 mg, Se 0.30 mg, Co 0.50 mg.

2 DE was a calculated value according to total energy and energy digestibility.

Each donkey was kept in a single stall (1.6 m × 2.0 m) and fed concentrate, corn silage and alfalfa at 7:00 a.m. and 2:00 p.m. every day. Millet straw was fed five times a day. Water was supplied ad libitum. Any leftover feed was collected each day in the morning before feeding, and the amount of feed offered was adjusted daily on the basis of the intake of the previous day to obtain the target refuse rate of 5–10%. The designated ratio of concentrate to forage was maintained. The feed offered and refused was weighed daily to calculate the daily matter intake (DMI) for each group.

### 2.4 Sample collection and analysis

#### 2.4.1 Milk sampling and analysis

From 7:00 a.m. to 10:00 a.m. and 2:00 p.m. to 5:00 p.m. every day, the donkeys were separated from their foals for 3 h each time, a total of 6 h per day, to allow for milk collection. Milking was conducted twice daily at 10:00 a.m. and 5:00 p.m. using an individual vacuum pumping machine (JuduH5402, Judu Technology, Hebei, China), and milk yield (MY, kg/d) was recorded at each milking, and daily MY was calculated by summing the two measurements (Li et al., 2013). Milk samples from the morning and afternoon were combined in a 1:1 ratio. One portion of this blended sample was analyzed for protein, fat, lactose, non-fat solids (SNF), and total solids (TS) using an automated mid-infrared milk analyzer (MilkoScan FT+, Foss Analytical Co., Ltd., Hillerød, Denmark). Another portion was preserved at −20 °C for subsequent analysis of FA. The estimated milk yield (EMY, kg/day) was calculated from MY measurements. Solids-corrected milk (SCM), milk production efficiency and milk protein synthesis efficiency were calculated according to the EMY and milk composition content. EMY (kg/day) = MY (kg/day)/isolation time (h) × 24 (h) (Liang et al., 2022). Milk production efficiency = SCM (kg/day)/DMI (kg/day) × 100. SCM (kg/day) = {(12.3 × milk fat (%) content of nonstandard milk + 6.56 × SNF (%) content of nonstandard milk − 0.0752)} × EMY (kg/day) (Yue et al., 2022). Milk protein synthesis efficiency = {EMY (kg/day) × milk protein (%)}/{DMI (kg/day) × CP level (%)} (Leiber et al., 2015).

FA methyl esters were produced from 1 g samples of Freeze-dried donkey milk powder following the method of Liang et al. (2025). For determination of FA concentration, a gas chromatograph (Agilent 7890B, DE, USA) with a SP™-2560 (Supelco, 100 m × 0.25 mm, 0.2 μm) was used. The injector temperature was 248 °C. The temperature program was configured as follows: initiate at 120 °C, maintain for 5 min, then escalate to 170 °C at a rate of 3 °C/min and hold for 10 min; further increase to 220 °C at 3 °C/min and maintain for 5 min; finally, elevate to 240 °C at 1 °C/min and hold for 10 min. Nitrogen served as the carrier gas, flowing at a rate of 3 mL/min with a split ratio of 9:1. The mixed standard for the FA methyl esters was sourced from Sigma Company.

The contents of 37 single FA were determined, and SFA, UFA, MUFA, PUFA, n-6 PUFA, n-3 PUFA, n-6/n-3, U/S, P/S, desirable fatty acid (DFA), atherogenicity index (AI), and thrombogenic index (TI) values were calculated. SFA in the milk including C4:0, C6:0, C8:0, C10:0, C11:0, C12:0, C13:0, C14:0, C15:0, C16:0, C17:0, C18:0, C20:0, C21:0, C22:0, C23:0 and C24:0. MUFA in the milk including C14:1, C15:1, C16:1, C17:1, C18:1t9, C18:1c9, C20:1, C22:1 and C24:1. n-6 PUFA including C18:2 t6, linoleic acid (C18:2c6), C18:3n6, C20:2n6, C20:3n6, C20:4n6 and C22:2n6. n-3 PUFA including C18:3n3, C20:3n3, eicosapentaenoic acid (C20:5n3) and C22:6n3. PUFA = n-3 PUFA + n-6 PUFA, n-6/n-3 = n-6 PUFA/n-3 PUFA, P/S = PUFA/SFA; UFA/SFA = U/S. DFA = C18:0 + UFA (Wereńska et al., 2021), AI = (C12:0 + (4 × C14:0) + C16:0)/UFA (Ulbricht and Southgate, 1991), TI = (C14:0 + C16:0 + C18:0)/[0.5 (MUFA) + 0.5 (n-6 PUFA) + 3 (n-3 PUFA) + (n-3 PUFA/n-6 PUFA)] (Ulbricht and Southgate, 1991), (C18:0 + C18:1)/C16:0 (Wereńska et al., 2021).

#### 2.4.2 Apparent nutrient digestion and metabolism

Before the start of the experiment, dietary samples were collected and dried in a 65 °C oven. After crushing and sifting, air-dried samples were obtained and stored in a dry, dark and ventilated place to determine nutrient composition. During the 8th week of the experiment, 200 g of rectal feces from each donkey were collected consistently over six consecutive days, then thoroughly mixed and divided into two parts. One portion was treated with H_2_SO_4_ (10%, v/v) to prevent nitrogen loss and subsequently stored at −20 °C for future nitrogen analysis. Another portion was directly dried at 65 °C to determine the DM (method 930.15), CP (method 984.13), ether extract (EE, method 920.39) based on AOAC International methods (Helrich, 2006). NDF and ADF were analyzed with an Ankom 220 Fiber Analyzer (Ankom Co., NY, USA) following the methods proposed by Van Soest et al. (1991). Acid-insoluble ash (AIA) was used to determine the apparent total-tract digestibility (ATTD) of a certain nutrient according to the description of Ren et al. (2020) using the following formula:


ATTD(%)=100−[(A1×B)/(A×B1)]×100.

Where A is the content of a given nutrient in the diet (%), A1 is the content of the same nutrient in the feces (%), B is the content of AIA in the diet (%), and B1 is the content of AIA in the feces (%).

A metabolic trial was conducted during the final 6 days of the experimental period using the methods outlined by Liang et al. (2022). Urine samples were transferred into Corning sample vessels (Corning Incorporated Costar, NY, USA). One sample remained untreated for creatinine and gross energy (GE) analysis, while the other was treated with 10 N sulfuric acid for nitrogen fixation to assess urinary nitrogen levels (Reynolds et al., 2014). All samples were stored at −20 °C.

GE in the diet, feces and urine samples was measured using an oxygen bomb calorimeter (6400 Automatic Analyzer Parr, IL, USA) according to the methods described by Jha et al. (2011). Energy digestibility and metabolisability were determined according to equations: energy digestibility = {diet GE (MJ/kg) × DMI -fecal GE (MJ/kg) × fecal output (kg)}/diet GE (MJ/kg) × DMI (kg) (Jha et al., 2011), and energy metabolisability = {diet GE (MJ/kg) × DMI (kg) - fecal GE (MJ/kg) × fecal output-urine GE (MJ/kg) × urine output (kg)}/diet GE (MJ/kg) × DMI (kg) (Jha et al., 2011); Fecal output = DMI × (AIA % in feed/AIA % in feces). The urine volume of the female donkeys was calculated according to the creatinine content in the urine, according to equation: Urine output = body weight (kg) × 24.05/creatinine (urine)/113 (Liang et al., 2022). N metabolisability = biological value (BV) × N digestibility, and BV = {N intake (kg/day)-N feces (kg/day)-N urine (kg/day)}/{N intake (kg/day)-N feces (kg/day)} × 100%.

#### 2.4.3 Blood sampling and analysis

Blood samples were collected from the jugular vein of all experimental donkeys before morning feeding on the last day of the experiment, using both ordinary blood collection tubes and heparin sodium blood collection tubes (Corning, Corning Incorporated Costar, NY, USA). The samples were centrifuged at 2,500×*g* for 15 min to separate the serum and plasma. Both fractions were subsequently stored at −20 °C pending further analysis. The FA content in the plasma was determined using an Agilent 7890B gas chromatograph (Agilent Technologies, DE, USA) following the methods described by Wang et al. (2020). Serum lipid metabolism-related enzyme activities, including fatty acid synthase (FAS), acetyl-CoA carboxylase (ACC), fatty acid elongase 5 (ELOVL5), fatty acid desaturase 1 (FADS1), lipoprotein lipase (LPL), hormone-sensitive lipase (HSL) and stearoyl-CoA desaturase (SCD), were determined using enzyme-linked immunosorbent assay kits (Shanghai yanjin Biotechnology Institute, Shanghai, China).

#### 2.4.4 Short-chain fatty acids analysis of the feces

During the final day of the experiment, rectal feces samples were collected from lactating donkeys using sterile gloves (2 collections per donkey). The samples were stored in DNase and RNase-free tubes (Shanghai Jingke Chemical Technology Co., Ltd., Shanghai), immediately frozen in liquid nitrogen (−196 °C), and reserved for subsequent analysis of short-chain fatty acids (SCFA). SCFA mainly include acetate, propionate, and butyrate. SCFA were produced from 1.5 g of rectal feces, as reported by Li et al. (2024). A Shimadzu 2014 gas chromatograph (Agilent Technologies, Santa Clara, CA) was utilized with nitrogen as the carrier gas, in conjunction with a DB-FFAP column (60 m × 0.25 mm × 0.5 μm). The temperatures of the column, detector, and injector were 120 °C, 250 °C, and 220 °C, respectively. The quantification of SCFA was completed by comparison against known standards (Supelco Volatile Fatty Acid Standard Mix, Sigma-Aldrich, St. Louis, MO, USA).

#### 2.4.5 Rectal microbiome analysis

During the final day of the experiment, rectal feces samples were collected from lactating donkeys using sterile gloves (2 collections per donkey). The samples were stored in DNase and RNase-free tubes (Shanghai Jingke Chemical Technology Co., Ltd., Shanghai), immediately frozen in liquid nitrogen (−196 °C), and reserved for 16S microbiome analysis. To extract DNA, fecal samples from each donkey were thawed on ice. The E.Z.N.A.® soil DNA Kit (Omega Bio-tek, Norcross, GA, U.S.) was used to extract the total microbial DNA from 14 rectal feces samples according to the manufacturer’s instructions. DNA purity and concentration were detected with NanoDrop2000 (Thermo Fisher, New York, USA) and DNA integrity was assessed by 1% agarose gel electrophoresis. The hypervariable region V3-V4 of the bacterial 16S rRNA gene were amplified with primer pairs 338F (5′-ACTCCTACGGGAGGCAGCAG-3′) and 806R (5′-GGACTACHVGGGTWTCTAAT-3′) by an ABI GeneAmp® 9700 PCR thermocycler (ABI, CA, USA). The PCR system (20 μL) included 4 μL 5 × FastPfu Buffer, 2 μL 2.5 mmol/L dNTPs, 0.8 μL forward primer (5 μmol/L), 0.8 μL reverse primer (5 μmol/L), 0.4 μL FastPfu Polymerase, 0.2 μL BSA, 10 ng template DNA, and ddH2O. Three PCR replicates for each sample were mixed, and 5 μL of the PCR products of each sample were detected by 2% agarose gel electrophoresis. The PCR product was extracted from 2% agarose gel and purified using the PCR Clean-Up Kit (YuHua, Shanghai, China) according to manufacturer’s instructions and quantified using Qubit 4.0 (Thermo Fisher Scientific, USA). Sequencing was conducted on the MiSeq PE300 platform (Illumina, San Diego, USA) according to the standard protocols by Majorbio Bio-Pharm Technology Co. Ltd. (Shanghai, China).

Quality control and adapter trimming were performed using fastp (version 0.19.6). Paired-end reads were merged using FLASH (version 1.2.7) with the following parameters: (i) reads were truncated at sites with an average quality score < 20 within a 50 bp sliding window, and those shorter than 50 bp or containing ambiguous bases were discarded; (ii) overlapping sequences longer than 10 bp were assembled with a maximum mismatch ratio of 0.2 in the overlapping region - reads that could not be assembled were discarded; (iii) barcode and primer matching was conducted using exact barcode matching and allowing up to 2 nucleotide mismatches in primer sequences. Processed sequences were clustered into OTU at 97% similarity using UPARSE (version 7.1).^1^ Taxonomic classification of representative OTU sequences was performed using the RDP Classifier (version 2.2) against the SILVA v138 16S rRNA database with a confidence threshold of 0.7. Subsequent analyses included alpha-diversity analysis,^2^ construction of Venn diagrams, community composition analysis, intergroup significance testing at the phylum and genus levels, LEfSe analysis, and calculation of Spearman rank correlation coefficients between environmental factors and prominent genera. The resulting numerical matrices were visualized as heatmaps, where colors indicate the magnitude of values within the matrix. Beta diversity was assessed through principal coordinate analysis (PCoA) based on Bray-Curtis distances. All statistical analyses and visualizations were implemented in R (version 3.3.1).

### 2.5 Statistical analysis

All statistical analyses were conducted using SAS software (version 8.1; SAS Institute Inc., Cary, NC, USA). A mixed-effects model was implemented using the PROC MIXED procedure to analyze repeated-measures data, including DMI, MY, EMY, SCM/DMI ratio, milk protein synthesis efficiency, milk composition profiles, and their corresponding yields. The model was specified as follows: Yijk = μ + Ci + Wj + Ci × Wj + bXjk + εijk, where Yijk = dependent variable; μ = overall mean; Ci = fixed effect of dietary AOCP levels, Wj = fixed effect of lactation week (weeks 1, 2, 3, 4, 5, 6, 7, and 8), Ci × Wj = effect of the interaction between diet treatment and lactation week, bXjk = effect of covariate (week 0, the observations during the 2 weeks of pretrial period served as covariates for the corresponding experimental period), εijk = residual error. The other parameters were analyzed using the T-Test procedure on normally distributed data, otherwise using the Kruskal-Wallis test. The differences in species composition at phylum and genus classification levels were analyzed using Kruskal-Wallis rank sum tests in SAS. Differentially abundant bacterial genera in the rectal microbiota were identified using LEfSe, with a significance threshold set at a logarithmic LDA score of > 3. The correlations between differential bacteria genera and FA composition, as well as nutrient digestibility and lactation performance, were calculated by Spearman’s correlation coefficient, respectively. Differences were considered statistically significant at *p* < 0.05 and a trend toward significance was considered at 0.05 ≤ *p* < 0.10.

## 3 Results

### 3.1 Fatty acid composition in plasma and milk

As shown in Table 2, compared with the CON group, the AOCP group significantly increased the proportions of C16:1 (*p* = 0.003), C18:1c9 (*p* = 0.006), C18:2c6 (*p* = 0.025), C20:3n6 (*p* = 0.003), C20:5n3 (*p* = 0.007), UFA (*p* = 0.024), PUFA (*p* = 0.026), n-3 PUFA (*p* = 0.028), and n-6 PUFA (*p* = 0.026), as well as the U/S (*p* = 0.026) and P/S (*p* = 0.026) ratios in plasma. DFA showed a trend of increase (*p* = 0.086). Conversely, the AOCP group significantly decreased the proportions of C21:0 (*p* = 0.003), C22:0 (*p* = 0.021), C24:0 (*p* = 0.023), C17:1 (*p* = 0.004), C22:1 (*p* < 0.001), C20:2n6 (*p* = 0.042), C22:2n6 (*p* < 0.001), C20:3n3 (*p* < 0.001), and SFA (*p* = 0.024) in plasma. Supplement: Except for the FAs mentioned above, the other FAs did not differ between the two groups (*p* > 0.05; see Supplementary Table S1).

**Table 2:** Effects of AOCP on partial fatty acid composition in blood of lactating donkeys (%, TFA)

Items^3^	CON^1^	AOCP^1^	SEM^2^	*P*-value
C11:0	0.13	0.10	0.028	0.390
C14:0	0.47	0.50	0.057	0.706
C16:0	15.34	16.31	0.497	0.198
C20:0	0.43	0.44	0.010	0.528
C21:0	0.06^a^	0.04^b^	0.004	0.003
C22:0	0.25^a^	0.21^b^	0.009	0.021
C24:0	0.36^a^	0.31^b^	0.012	0.023
C16:1	0.66^b^	0.77^a^	0.021	0.003
C17:1	0.30^a^	0.24^b^	0.011	0.004
C18:1t9	0.08	0.08	0.008	0.723
C18:1c9	10.42^b^	11.49^a^	0.215	0.006
C20:1	0.36	0.35	0.052	0.266
C22:1	4.75^a^	3.77^b^	0.112	<0.001
C18:2t6	0.04	0.03	0.004	0.175
C18:2c6	38.14^b^	41.49^a^	0.899	0.025
C20:2n6	0.34^a^	0.31^b^	0.007	0.042
C20:3n6	0.13^b^	0.15^a^	0.002	0.003
C22:2n6	0.16^a^	0.13^b^	0.004	<0.001
C20:3n3	0.17^a^	0.13^b^	0.005	<0.001
C20:5n3	0.07^b^	0.10^a^	0.007	0.007
SFA	42.98^a^	39.52^b^	0.895	0.024
UFA	57.02^b^	60.49^a^	0.895	0.024
MUFA	17.16	17.31	0.220	0.665
PUFA	39.86^b^	43.18^a^	0.895	0.026
n-3 PUFA	0.98^b^	1.02^a^	0.008	0.028
n-6 PUFA	38.89^b^	42.19^a^	0.897	0.026
n-6/n-3	39.59	40.49	0.720	0.428
U/S	1.33^b^	1.54^a^	0.054	0.026
P/S	0.93^b^	1.10^a^	0.044	0.026
Desirable fatty acid (DFA)	76.25	79.16	1.040	0.086
Atherogenicity Index (AI)	0.30	0.30	0.014	0.988
Thrombogenic Index (TI)	1.13	1.08	0.027	0.220
(C18:0 + C18:1)/C16:0	1.95	1.86	0.053	0.292

^a,b^Means within a row with different letters are significantly different (*P* < 0.05).

1 CON = control (basal diet); AOCP = *Artemisia ordosica* crude polysaccharides (basal diet with 0.5 g/kg DM AOCP).

2 SEM = standard error of the mean.

3 SFA = saturated fatty acid (6:0 + 8:0 + 10:0 + 11:0 + 12:0 + 13:0 + 14:0 + 15:0 + 16:0 + 17:0 + 18:0 + 20:0 + 21:0 + 22:0 + 23:0 + 24:0); UFA = unsaturated fatty acid, includes MUFA (monounsaturated fatty acids) and PUFA (polyunsaturated fatty acids); MUFA (14:1 + 15:1 + 16:1 + 17:1 + 18:1t9 + 18:1c9 + 20:1 + 22:1 + 24:1); PUFA (n-6 PUFA + n-3 PUFA); n-6 PUFA (18:2t6 + 18:2c6 + 18:3n6 + 20:2n6 + 20:3n6 + 20:4n6 + 22:2n6); n-3 PUFA (18:3n3 + 20:3n3 + 20:5n3 + 22:6n3); n-6/n-3 = n-6 PUFA/n-3 PUFA ratio; U/S = UFA to SFA ratio; P/S = PUFA to SFA ratio.

The effects of AOCP supplementation on milk FA composition and yield are presented in Table 3. Compared with the CON group, the AOCP group significantly increased proportions and yields of C16:0 (*p* = 0.026; *p* = 0.039), C16:1 (*p* = 0.001; *p* = 0.005), C18:1 t9 (*p* = 0.008; *p* = 0.037), C18:1c9 (*p* = 0.043; *p* = 0.042), C20:1 (*p* = 0.009; *p* = 0.011), C20:5n3 (*p* < 0.001; *p* < 0.001), UFA (*p* = 0.002; *p* = 0.040), MUFA (*p* = 0.018; *p* = 0.027), and DFA (*p* = 0.007; *p* = 0.043), as well as the U/S (*p* = 0.009; *p* = 0.011) and P/S (*p* = 0.015; *p* = 0.042). The AOCP group also showed increased proportions of C18:2c6 (*p* = 0.043), PUFA (*p* = 0.024), and n-6 PUFA (*p* = 0.047), along with increased yields of C20:3n6 (*p* = 0.019). The proportion of C20:0 (*p* = 0.097) and the yields of C18:2t6 (*p* = 0.059), C18:2c6 (*p* = 0.087), and n-6 PUFA (*p* = 0.093) showed an increasing trend. Conversely, the AOCP group significantly decreased proportions of C11:0 (*p* = 0.003), C14:0 (*p* < 0.001), C22:1 (*p* = 0.010), C20:2n6 (*p* = 0.021), C20:3n3 (*p* = 0.001), SFA (*p* = 0.002), and AI (*p* = 0.007), as well as decreased yields of C11:0 (*p* = 0.002) and AI (*p* = 0.007). Among the other FAs listed in Supplementary Table S2, only the proportion of C6:0 (*p* = 0.086) showed an increasing trend, while no significant differences were observed for the others (*p* > 0.05)

**Table 3:** Effects of AOCP on partial fatty acid composition and yield in milk of lactating donkeys

Items^3^	milk FA composition (%, TFA)				milk FA yield (g/day)			
	CON^1^	AOCP^1^	SEM^2^	*P*-value	CON^1^	AOCP^1^	SEM^2^	*P*-value
C11:0	0.03^a^	0.01^b^	0.002	0.003	0.11^a^	0.05^b^	0.005	0.002
C14:0	6.69^a^	6.06^b^	0.073	<0.001	24.76	23.66	0.984	0.443
C16:0	18.51^b^	20.14^a^	0.454	0.026	68.39^b^	78.69^a^	2.994	0.039
C20:0	0.03	0.04	0.001	0.097	0.13	0.15	0.008	0.124
C21:0	0.01	0.01	0.004	0.476	0.05	0.03	0.018	0.767
C22:0	0.01	0.01	0.001	0.669	0.05	0.05	0.003	0.930
C24:0	0.00	0.00	0.001	0.783	0.02	0.02	0.005	0.370
C16:1	2.17^b^	2.67^a^	0.068	0.001	8.06^b^	10.43^a^	0.473	0.005
C17:1	0.01	0.01	0.001	0.072	0.02	0.03	0.005	0.139
C18:1t9	0.05^b^	0.06^a^	0.002	0.008	0.17^b^	0.22^a^	0.012	0.037
C18:1c9	18.05^b^	20.02^a^	0.597	0.043	66.90^b^	78.02^a^	3.369	0.042
C20:1	0.21^b^	0.25^a^	0.006	0.009	0.79^b^	0.96^a^	0.039	0.011
C22:1	0.09^a^	0.08^b^	0.003	0.010	0.33	0.29	0.017	0.166
C18:2t6	0.01	0.01	0.001	0.110	0.03	0.04	0.003	0.059
C18:2c6	17.41^b^	18.84^a^	0.442	0.043	64.45	73.95	3.530	0.087
C20:2n6	0.43^a^	0.40^b^	0.005	0.021	1.57	1.57	0.078	0.966
C20:3n6	0.04	0.04	0.001	0.102	0.13^b^	0.16^a^	0.008	0.019
C22:2n6	0.03	0.03	0.001	0.113	0.13	0.11	0.008	0.323
C20:3n3	0.10^a^	0.09^b^	0.001	0.001	0.36	0.34	0.017	0.407
C20:5n3	0.01^b^	0.02^a^	0.001	<0.001	0.02^b^	0.06^a^	0.002	<0.001
SFA	57.78^a^	53.84^b^	0.701	0.002	213.91	211.05	9.792	0.841
UFA	42.22^b^	46.16^a^	0.701	0.002	156.39^b^	180.50^a^	7.340	0.040
MUFA	20.82^b^	23.32^a^	0.618	0.018	77.18^b^	90.89^a^	3.755	0.027
PUFA	21.40^b^	22.84^a^	0.468	0.024	79.20	89.61	4.263	0.114
n-3 PUFA	3.46	3.50	0.125	0.820	12.80	13.69	0.734	0.408
n-6 PUFA	17.94^b^	19.34^a^	0.442	0.047	66.40	75.91	3.610	0.093
n-6/n-3	5.24	5.53	0.165	0.281	5.24	5.53	0.165	0.282
U/S	0.74^b^	0.85^a^	0.025	0.009	0.73^b^	0.86^a^	0.024	0.011
P/S	0.38^b^	0.42^a^	0.113	0.015	0.37^b^	0.43^a^	0.014	0.042
Desirable fatty acid (DFA)	43.83^b^	47.79^a^	0.684	0.007	162.35^b^	186.90^a^	7.609	0.043
Atherogenicity Index (AI)	1.31^a^	1.16^b^	0.030	0.007	1.31^a^	1.16^b^	0.030	0.007
Thrombogenic Index (TI)	0.90	0.87	0.027	0.544	0.90	0.88	0.027	0.502
(C18:0 + C18:1)/C16:0	1.07	1.08	0.030	0.775	1.07	1.08	0.030	0.775

^a,b^Means within a row with different letters are significantly different (*P* < 0.05).

1 CON = control (basal diet); AOCP = *Artemisia ordosica* crude polysaccharides (basal diet with 0.5 g/kg DM AOCP).

2 SEM = standard error of the mean.

SFA = saturated fatty acid (6:0 + 8:0 + 10:0 + 11:0 + 12:0 + 13:0 + 14:0 + 15:0 + 16:0 + 17:0 + 18:0 + 20:0 + 21:0 + 22:0 + 23:0 + 24:0); UFA = unsaturated fatty acid, includes MUFA (monounsaturated fatty acids) and PUFA (polyunsaturated fatty acids); MUFA (14:1 + 15:1 + 16:1 + 17:1 + 18:1t9 + 18:1c9 + 20:1 + 22:1 + 24:1); PUFA (n-6 PUFA + n-3 PUFA); n-6 PUFA (18:2t6 + 18:2c6 + 18:3n6 + 20:2n6 + 20:3n6 + 20:4n6 + 22:2n6); n-3 PUFA (18:3n3 + 20:3n3 + 20:5n3 + 22:6n3); n-6/n-3 = n-6 PUFA/n-3 PUFA ratio; U/S = UFA to SFA ratio; P/S = PUFA to SFA ratio.

### 3.2 Milk performance

As shown in Table 4, compared with the CON group, the DMI (*p* < 0.001), MY (*p* < 0.001), EMY (*p* < 0.001), milk fat (*p* = 0.005), lactose (*p* < 0.001), SNF (*p* = 0.020), and TS (*p* = 0.001) contents in the AOCP group were increased. Additionally, yields of milk fat (*p* = 0.025), protein (*p* = 0.033), lactose (*p* < 0.001), SNF (*p* = 0.007), and TS (*p* = 0.010) also significantly increased. No significant differences were observed between the two groups in milk production efficiency, milk protein synthesis efficiency, or milk protein content (*p >* 0.05).

**Table 4:** Effects of AOCP on DMI, milk production and components of lactating donkeys.

Items^3^	Treatment		SEM^2^	*p*-Value		
	CON^1^	AOCP^1^		Treatment	Week	Treatment*week
DMI (kg/day)	7.58^b^	8.00^a^	0.172	<0.001	<0.001	0.377
MY (kg/day)	0.86^b^	0.90^a^	0.013	<0.001	0.026	0.789
EMY (kg/day)	3.46^b^	3.61^a^	0.051	<0.001	0.022	0.744
Milk production efficiency	0.25	0.26	0.007	0.304	<0.001	0.768
Milk protein synthesis efficiency	0.79	0.82	0.018	0.163	<0.001	0.987
Milk components						
Fat (%)	0.37^b^	0.40^a^	0.009	0.005	0.003	0.003
Protein (%)	1.74	1.76	0.035	0.505	<0.001	0.298
Lactose (%)	7.10^b^	7.11^a^	0.018	<0.001	0.648	0.914
SNF (%)	8.86^b^	8.95^a^	0.027	0.020	<0.001	0.047
TS (%)	9.30^b^	9.34^a^	0.017	0.001	<0.001	0.221
Milk components yield						
Fat yield (g/day)	12.85^b^	14.35^a^	0.347	0.025	<0.001	0.005
Protein yield (g/day)	60.17^b^	63.67^a^	1.151	0.033	<0.001	0.395
Lactose yield (g/day)	245.74^b^	256.24^a^	4.230	<0.001	0.030	0.785
SNF yield (g/day)	306.44^b^	322.18^a^	4.393	0.007	<0.001	0.741
TS yield (g/day)	321.08^b^	336.99^a^	4.754	0.010	<0.001	0.690

^a,b^Means within a row with different letters are significantly different (*p* < 0.05).

1 CON, control (basal diet); AOCP, *Artemisia ordosica* crude polysaccharides (basal diet with 0.5 g/kg DM AOCP).

2 SEM, standard error of the mean.

3 DMI, dry matter intake; MY, milking yield; EMY, estimated milk yield; SNF, solid not fat; TS, total solids.

### 3.3 Nutrient digestibility

As shown in Table 5, the apparent digestibility of DM (*p* = 0.016), EE (*p* = 0.002), NDF (*p* = 0.029), ADF (*p* = 0.005) and energy metabolic rate (*p* = 0.010) increased in the AOCP group compared with the CON group. No significant differences in the apparent digestibility of CP, Ca, and P, as well as in energy digestibility, BV, and nitrogen metabolic rate existed between the CON and AOCP groups (*p* > 0.05).

**Table 5:** Effects of AOCP on apparent total-tract nutrient digestibility, energy and protein metabolic ratio of lactating donkeys.

Items^3^	CON^1^	AOCP^1^	SEM^2^	*p*-Value
DM (%)	66.02^b^	68.11^a^	0.518	0.016
CP (%)	80.42	80.43	0.519	0.987
EE (%)	79.26^b^	82.56^a^	0.523	0.002
NDF (%)	39.69^b^	42.11^a^	0.662	0.029
ADF (%)	33.48^b^	36.57^a^	0.610	0.005
Ca (%)	65.74	65.80	0.637	0.959
P (%)	16.72	20.67	1.888	0.165
Energy digestibility (%)	70.39	70.43	0.714	0.973
Energy metabolic rate (%)	38.85b	48.22^a^	2.098	0.010
Protein biological value (%)	71.45	73.48	1.221	0.285
Nitrogen metabolic rate (%)	57.46	59.11	1.096	0.322

^a,b^Means within a row with different letters are significantly different (*p* < 0.05).

1 CON, control (basal diet); AOCP, *Artemisia ordosica* crude polysaccharides (basal diet with 0.5 g/kg DM AOCP).

2 SEM, standard error of the mean.

3 ADF, acid detergent fiber; CP, crude protein; DM, dry matter; EE, ether extract; NDF, neutral detergent fiber.

### 3.4 Enzyme activities related to lipid metabolism in the serum

As shown in Table 6, compared with the CON group, the SCD (*p* < 0.001) activities was increased in the AOCP group, while the activities of ACC (*p* = 0.003) and HSL (*p* < 0.001) were declined. No significant differences in the activities of LPL, FAS, FADS1, and ELOVL5 between the two groups (*p* > 0.05).

**Table 6:** Effects of AOCP on serum enzyme activities related to lipid metabolism in lactating donkeys.

Items^3^	CON^1^	AOCP^1^	SEM^2^	*p*-value
ACC (U/L)	43.66^a^	37.48^b^	1.115	0.003
LPL (U/L)	497.86	476.79	13.866	0.306
HSL (U/L)	1,501.14^a^	1,079.00^b^	44.177	<0.001
FAS (U/mL)	1,224.29	1,238.57	17.997	0.629
SCD (U/L)	117.63^b^	149.33^a^	3.674	<0.001
FADS1 (U/L)	33.07	37.38	1.278	0.114
ELOVL5 (U/L)	76.03	72.9	2.579	0.479

^a,b^Means within a row with different letters are significantly different (*p* < 0.05).

^1^CON, control (basal diet); AOCP, *Artemisia ordosica* crude polysaccharides (basal diet with 0.5 g/kg DM AOCP).

^2^SEM, standard error of the mean.

^3^ACC, Acetyl CoA carboxylase; LPL, lipoprteinlipase; HSL, hormone-sensitive triglyceride lipase; FAS, fatty acid synthase; SCD, Stearoyl-CoA Desaturase; FADS1, Fatty Acid Desaturase 1; ELOVL5, elongase of verylong chain fatty acid 5.

### 3.5 Short-chain fatty acids in the rectal feces

As shown in Table 7, acetate (*p* = 0.002), butyrate (*p* = 0.002), total VFA (*p* = 0.003) levels and acetate-to-propionate (*p* < 0.001) ratio increased in the AOCP group, compared with the CON group. No significant difference was observed in propionate concentration between the CON and AOCP groups (*p* > 0.05).

**Table 7:** Effects of AOCP on SCFA of rectal feces in lactating donkeys.

Items	CON^1^	AOCP^1^	SEM^2^	*p*-Value
Acetate (mmol/L)	6.10^b^	6.65^a^	0.094	0.002
Propionate (mmol/L)	2.41	2.38	0.014	0.122
Butyrate (mmol/L)	0.82^b^	0.87^a^	0.009	0.002
Acetate: Propionate ration	2.53^b^	2.80^a^	0.036	<0.001
Total VFA (mmol/L)	9.98^b^	10.57^a^	0.109	0.003

^a,b^Means within a row with different letters are significantly different (*p* < 0.05).

1 CON, control (basal diet); AOCP, *Artemisia ordosica* crude polysaccharides (basal diet with 0.5 g/kg DM AOCP).

2 SEM, standard error of the mean.

### 3.6 Fecal bacterial richness, diversity and composition

**Figure 1:**
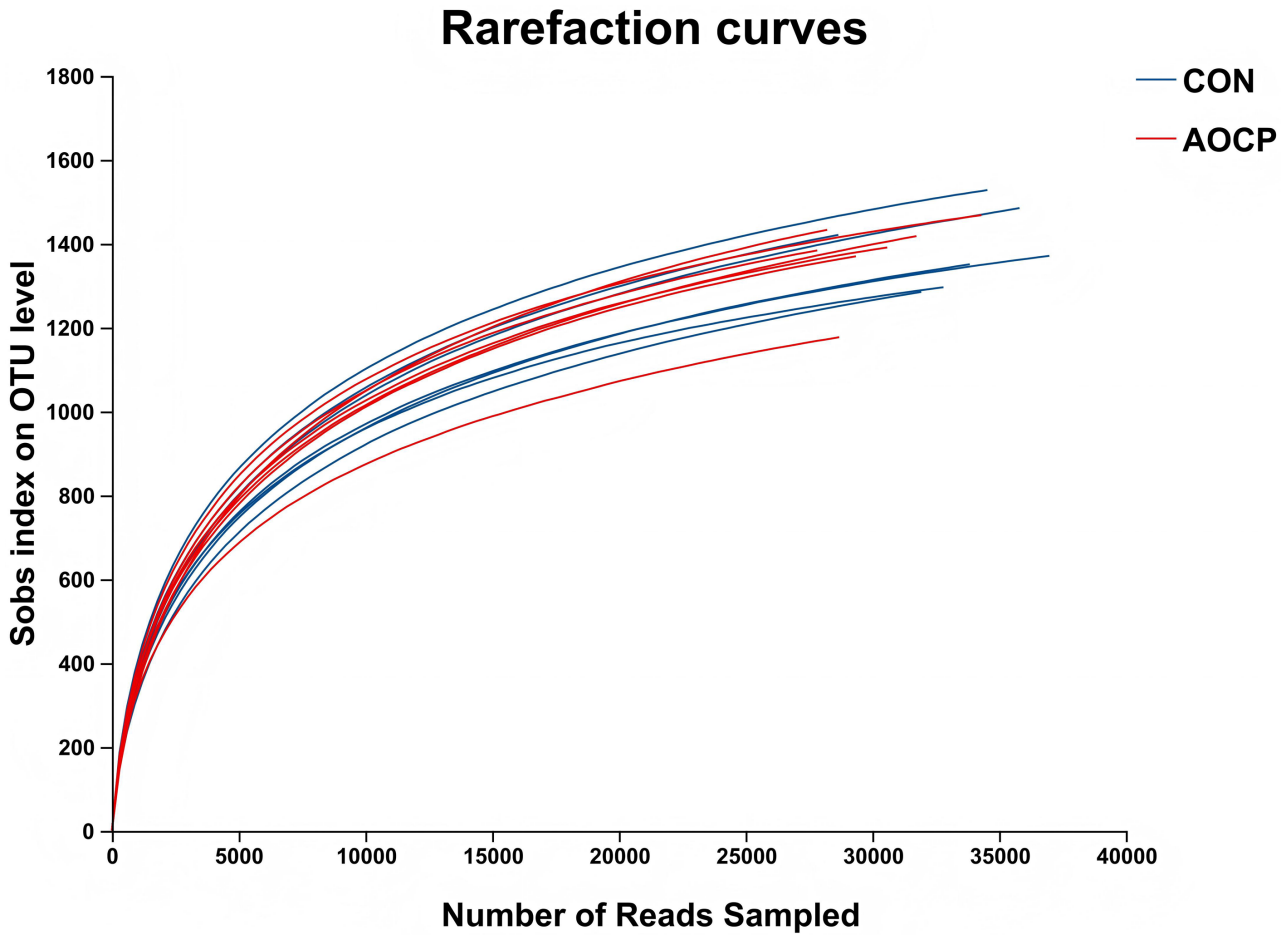
Rarefaction curves of OTU number in rectal microbiota. CON, control (basal diet); AOCP, *Artemisia ordosica* crude polysaccharide (basal diet with 0.5 g/kg DM AOCP).

A total of 684,335 effective 16S rRNA sequences were retrieved from 14 sequenced samples and 1980 OTUs were obtained by performing OTU clustering on nonrepetitive sequences according to 97% similarity. Rarefaction curves (Figure 1) showed that the current sequencing depth and sample size were sufficient to assess the microbial diversity, total species richness and core species number of rectal bacterial samples.

**Figure 2:**
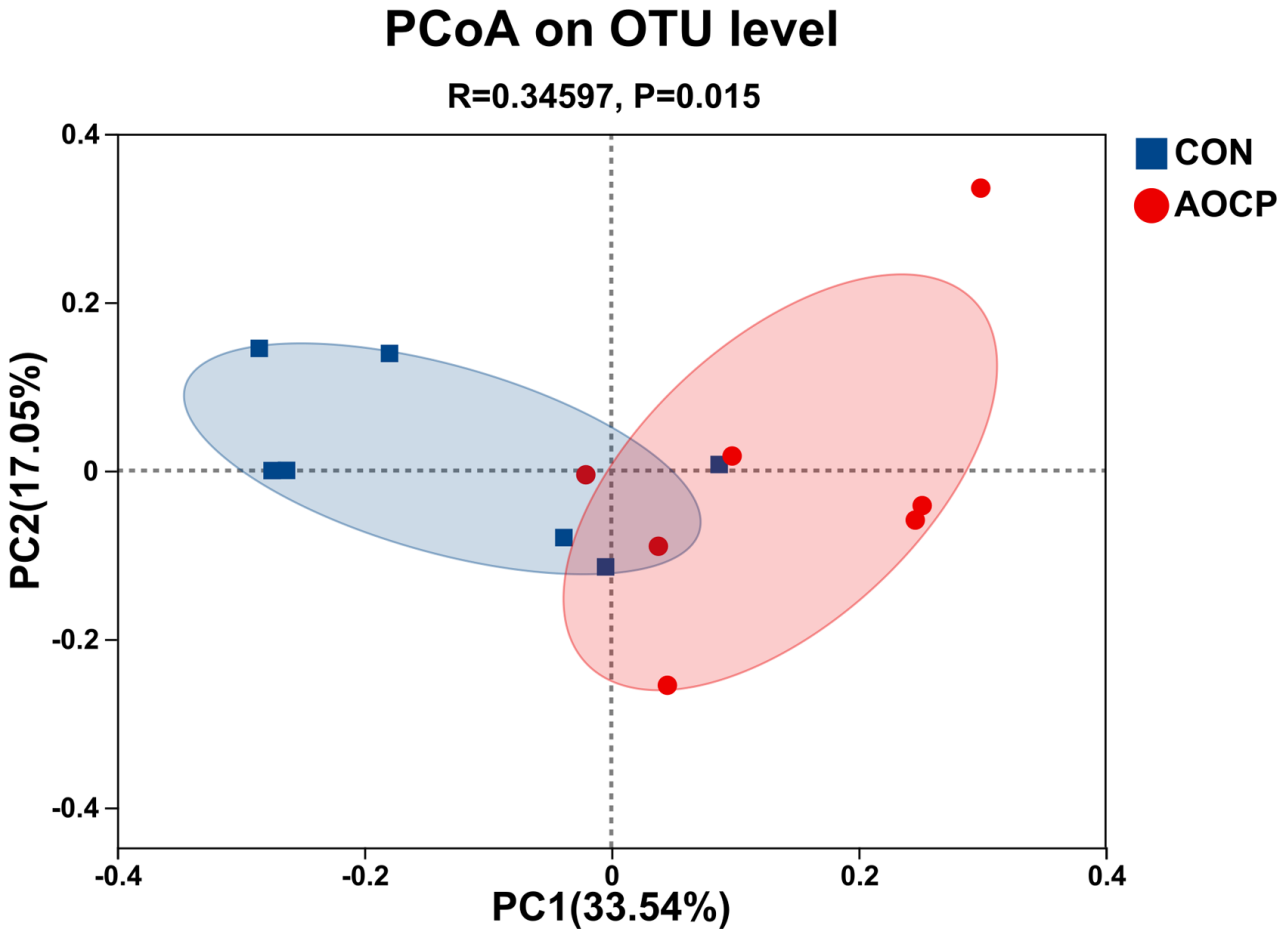
Beta diversity analysis of rectal microbiota via principal coordinate analysis (PCoA). CON, control (basal diet); AOCP, *Artemisia ordosica* crude polysaccharide (basal diet with 0.5 g/kg DM AOCP).

**Figure 3:**
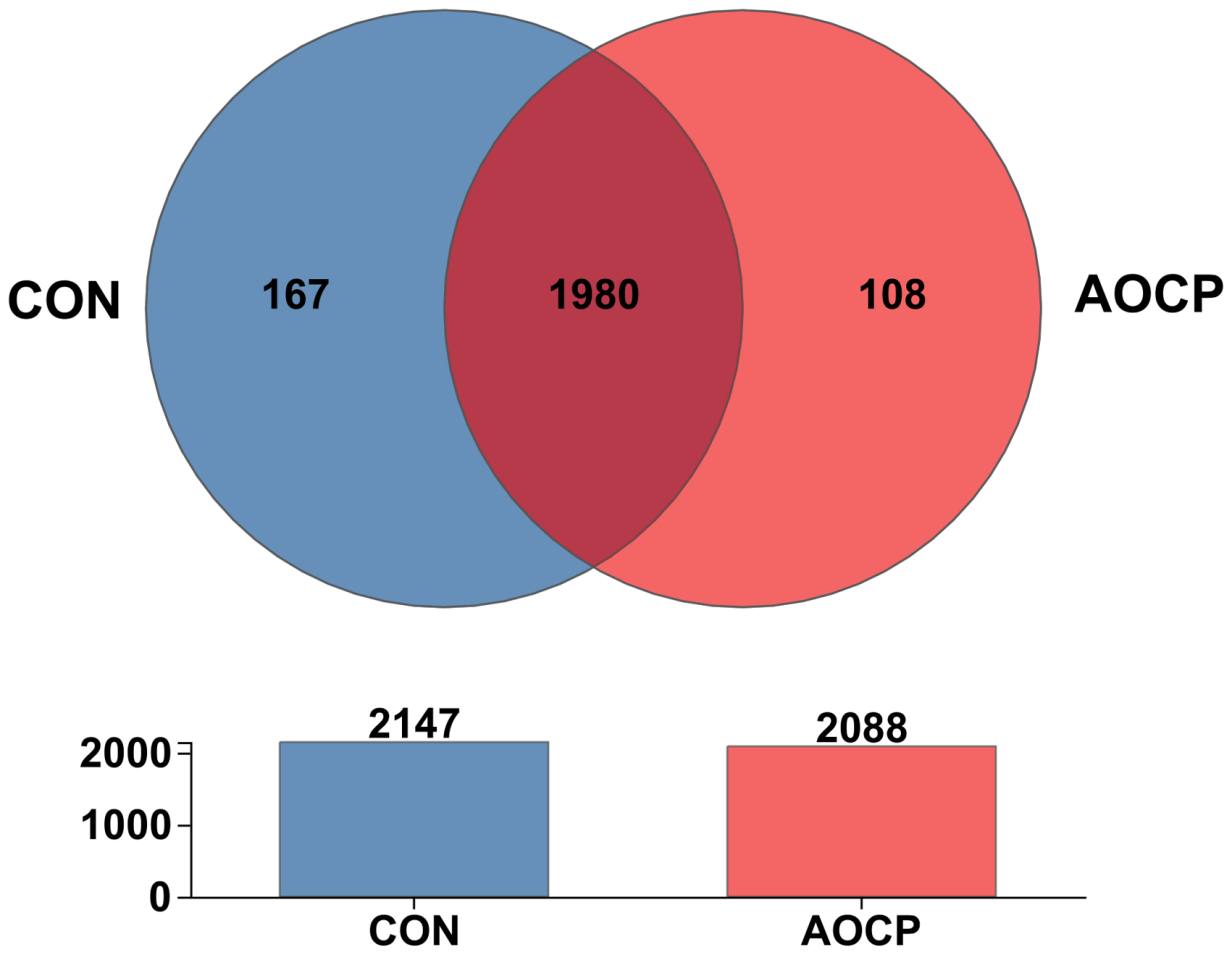
Common and unique OTU number of rectal bacteria in the control group and AOCP group. CON, control (basal diet); AOCP, *Artemisia ordosica* crude polysaccharide (basal diet with 0.5 g/kg DM AOCP).

The α-diversity analysis (Supplementary Table S3) revealed that the Shannon index was significantly higher in the AOCP group (5.69) than in the CON group (5.56) (*p* = 0.010), indicating that AOCP supplementation enhanced the diversity of the rectal microbial community. AOCP supplementation had no statistically significant impact on the α-diversity indices (Chao1, ACE, and Simpson) of the fecal microbiota compared with the CON group (*p* > 0.05), although certain numerical differences were noted. Specifically, both the Chao1 and ACE indices were higher in the AOCP group (1,600.77 and 1,598.07, respectively) than in the CON group (1,580.66 and 1,590.17 respectively). The coverage index of the sequencing results in each group reached 0.99, indicating that the coverage was sufficient to meet the requirements of subsequent analysis. The β-diversity analysis was performed to explore differences in the rectum microbial community between the two groups (Figure 2). The PCoA based on the Bray-Curtis distance matrix showed that, at the genus level, the points representing rectum microorganisms in the two groups, respectively, were significantly separated in different quadrants on the coordinate axis, which indicated that AOCP supplementation had a distinct effect on rectum microbial species and abundance. By performing a Venn diagram on the OTU samples with 97% identity, 167 and 108 unique OTUs were identified in the CON and AOCP groups, respectively (Figure 3).

### 3.7 Significantly different rectal bacteria between the CON and AOCP groups

We analyzed changes in rectal bacterial communities between the two groups at the phylum and genus levels to characterize the effects of AOCP on rectal bacterial community structure in donkeys. As shown in Table 8, at the phylum level, the rectal flora primarily consisted of *Firmicutes*, *Bacteroidota*, *Spirochaetot*a, *Verrucomicrobiota*, and *Actinobacteriota*. AOCP increased the relative abundance of phylum *Bacteroidota* (*p* < 0.05), and decreased the relative abundance of phylum *Firmicutes* (*p* < 0.05).

**Table 8:** Effects of AOCP on the composition of rectal bacteria at phyla level (more than 1% of total bacteria).

Items	CON^1^	AOCP^1^	SEM^2^	*p*-Value
Firmicutes	68.22^a^	58.96^b^	1.607	0.004
Bacteroidota	18.73^b^	28.51^a^	1.177	0.001
Spirochaetota	6.01	6.10	1.017	0.956
Verrucomicrobiota	3.21	3.48	0.346	0.691
Actinobacteriota	1.07	1.06	0.093	0.921

1 CON, control (basal diet); AOCP, *Artemisia ordosica* crude polysaccharides (basal diet with 0.5 g/kg DM AOCP).

2 SEM, standard error of the mean.

**Figure 4:**
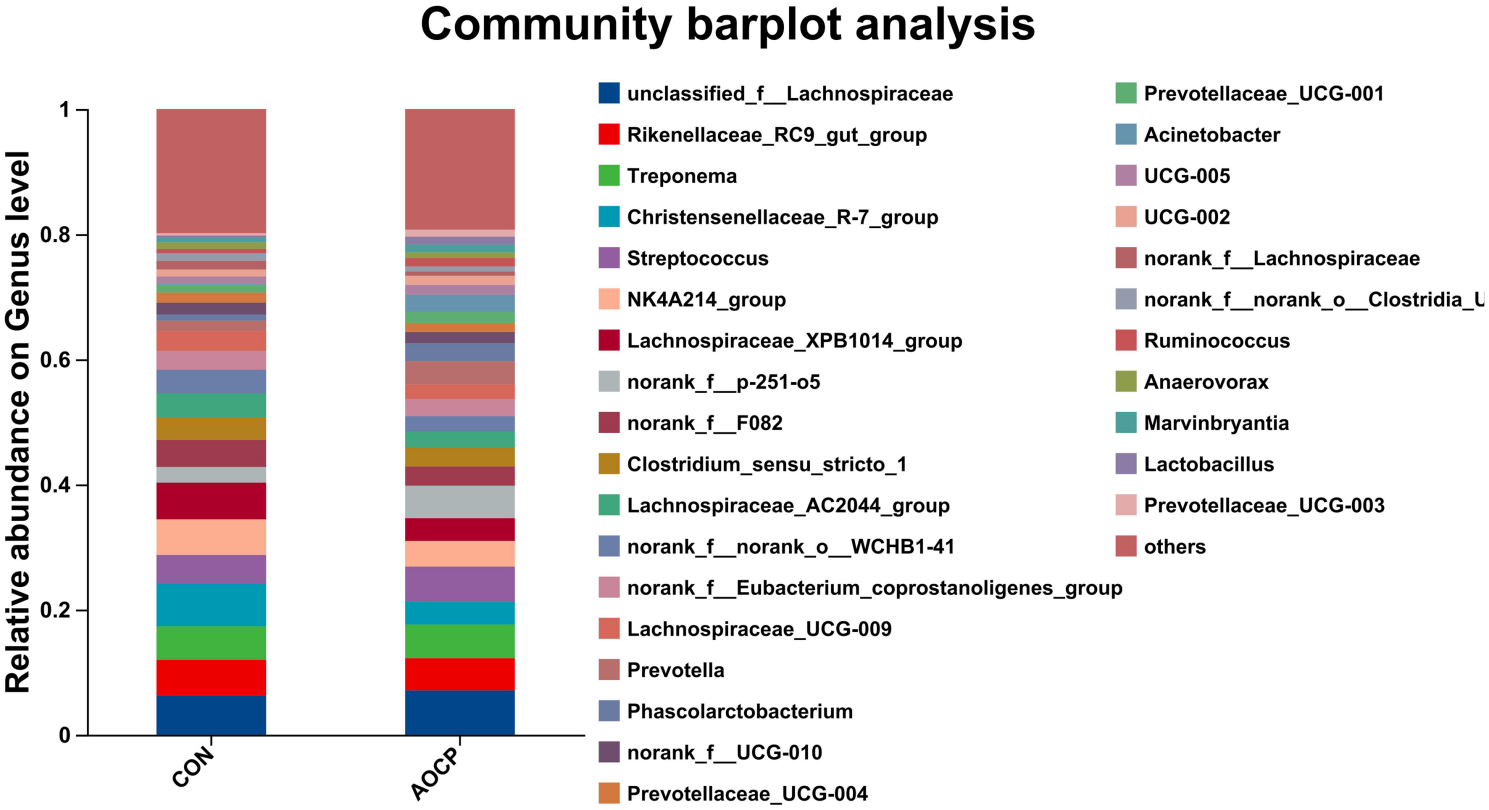
Community compositions of rectal bacteria at the genus level in the control and AOCP groups. CON, control (basal diet); AOCP, *Artemisia ordosica* crude polysaccharide (basal diet with 0.5 g/kg DM AOCP). The different colors of the bars represent different species, and the length of the bars represents the proportion of the species.

**Figure 5:**
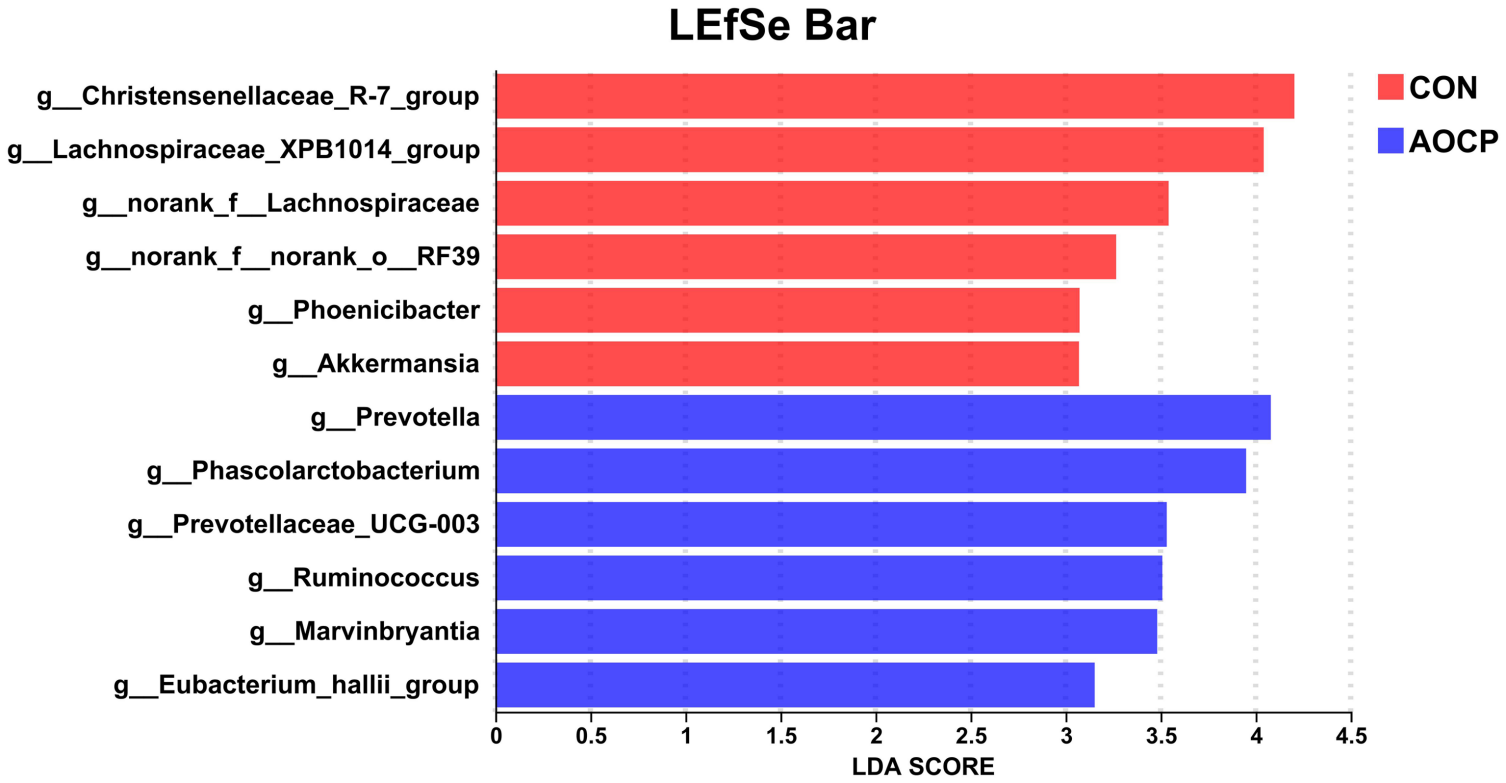
LEfSe analysis of differentially abundant rectal bacterial taxa between the CON and AOCP groups, using a threshold LDA score > 3 for statistical significance. CON, control (basal diet); AOCP, *Artemisia ordosica* crude polysaccharide (basal diet with 0.5 g/kg DM AOCP).

As shown in Table 9 and Figure 4, the top 20 bacterial genera (relative abundance >1%) were analyzed at the genus level, with *unclassified_f_Lachnospiraceae* being the most dominant genus. Compared to the CON group, AOCP supplementation significantly increased the abundance of *unclassified_f_Lachnospiraceae* and *Prevotella* (*p* < 0.05), while reducing the abundance of *Christensenellaceae_R-7_group*, *Streptococcus* and *Lachnospiraceae_XPB1014* (*p* < 0.05). Furthermore, the significantly different abundant bacteria in the rectum between the two groups were identified through LEfSe analysis (Figure 5). The relative abundances of *Prevotella*, *Phascolarctobacterium*, *Prevotellaceae_UCG-003*, *Ruminococcus*, *Marvinbryantia*, and *Eubacterium_hallii_group* were increased in the AOCP group compared with the CON group (*p* < 0.05). In contrast, *Christensenaceae_R-7*, *Lachnospiraceae_XPB1014_group*, *norank_f_Lachnospiraceae*, *norank_f_norank_o_RF39*, *Phoenicibacter*, *Akkermansi*a were decreased in the rectum with the AOCP supplementation (*p* < 0.05).

**Table 9:** Effects of AOCP on the composition of rectal bacteria at genus level (more than 1% of total bacteria).

Phylum	Genus	Treatments		SEM^2^	*p*-value
		CON^1^	AOCP^1^		
*Firmicutes*	*unclassified_f_Lachnospiraceae*	5.90^b^	7.66^a^	0.352	0.008
	*Christensenellaceae_R-7*	8.95^a^	2.94^b^	0.279	<0.001
	*Streptococcus*	5.76^a^	4.53^b^	0.198	0.001
	*Oscillospiraceae__NK4A214*	4.35	3.49	0.287	0.163
	*Lachnospiraceae_XPB1014*	5.35^a^	3.33^b^	0.357	0.015
	*Clostridium_sensu_stricto_1*	4.14	4.07	0.443	0.946
	*Lachnospiraceae_AC2044*	3.22	3.06	0.326	0.811
	*norank_f__Eubacterium_coprostanoligenes*	3.27	3.96	0.297	0.559
	*Lachnospiraceae_UCG-009*	2.56	2.00	0.224	0.151
	*norank_f___UCG-010*	1.92	1.82	0.18	0.685
	*UCG-005*	1.30	1.42	0.146	0.627
	*UCG-002*	1.00	1.14	0.149	0.563
*Bacteroidota*	*Rikenellaceae_RC9_gut*	5.47	5.57	0.302	0.838
	*norank_f__F082*	3.74	3.09	0.384	0.296
	*Prevotella*	1.07^b^	4.04^a^	0.242	<0.001
	*Prevotellaceae_UCG-004*	1.75	1.66	0.238	0.804
	*Prevotellaceae_UCG-001*	1.19	1.40	0.216	0.514
*Spirochaetota*	*Treponema*	4.62	4.26	0.631	0.771
*Verrucomicrobiota*	*norank_f__norank_o__WCHB1-41*	2.54	2.17	0.286	0.432
*Actinobacteriota*	*norank_f__p-251-o*	1.89	1.68	0.275	0.699

1 CON, control (basal diet); AOCP, *Artemisia ordosica* crude polysaccharides (basal diet with 0.5 g/kg DM AOCP).

2 SEM, standard error of the mean.

### 3.8 Correlation analysis of different rectal bacterial genera with milk fatty acid composition, nutrient digestibility, and lactation performance

**Figure 6:**
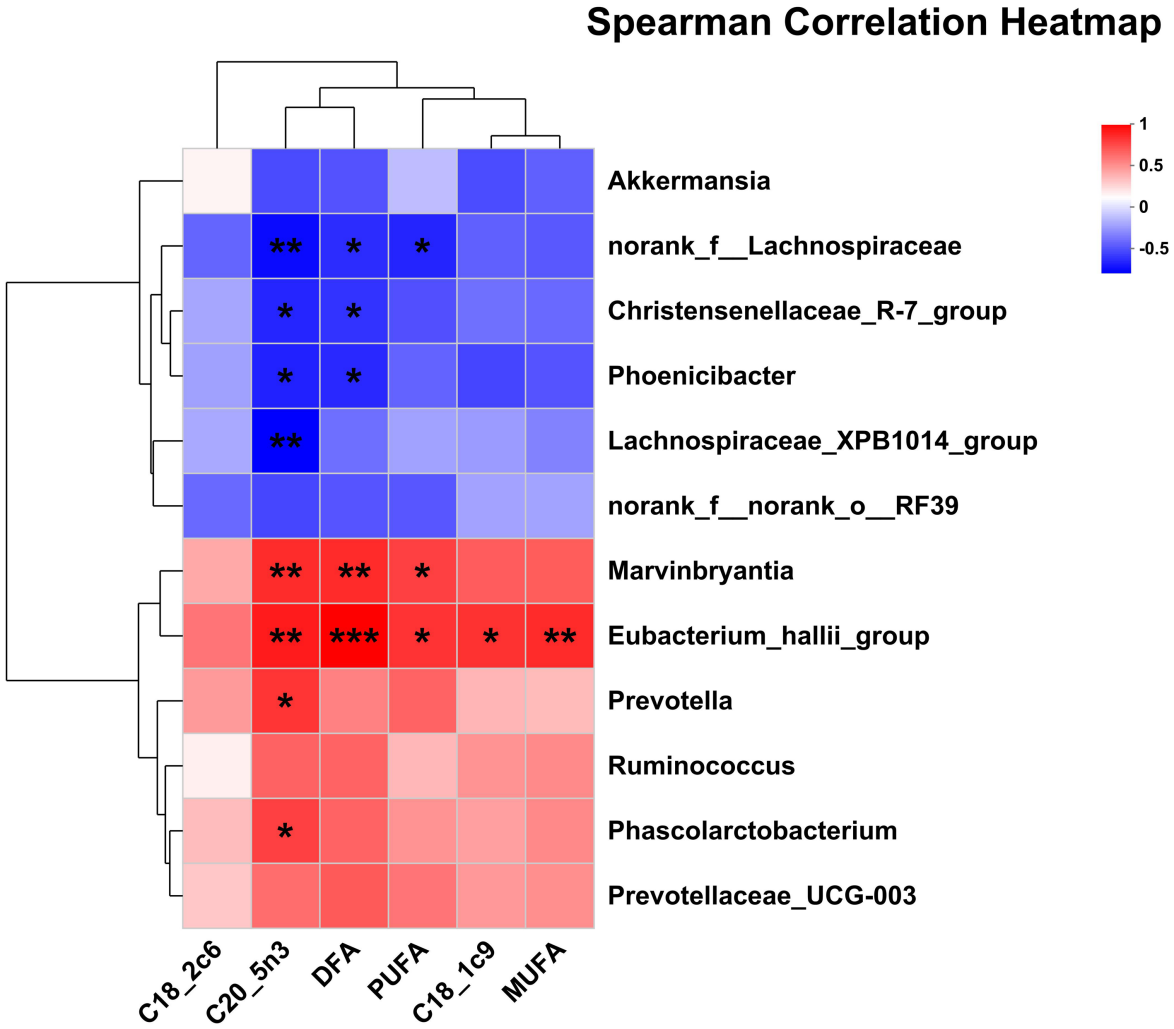
Pearson correlation analysis of differential bacteria genus and milk FA composition. Red indicates a positive correlation; the blue indicates a negative correlation. ***p ≤ 0.001, **0.001 < *p* ≤ 0.01, *0.01 < *p* ≤ 0.05. DFA, desirable fatty acid (C18:0 + UFA).

The correlations between the significantly differential rectal bacteria and milk FA composition are presented in Figure 6. The proportion of C18:1c9 and MUFA was positively correlated with *Eubacterium_hallii_group*. The proportion of PUFA was negatively correlated with *norank_f__Lachnospiraceae* but was positively correlated with *Marvinbryantia*, *Eubacterium_hallii_group*. The proportion of DFA was negatively correlated with *Phoenicibacter*, *norank_f__Lachnospiraceae*, *Christensenellaceae_R-7_group*, but was positively correlated with *Eubacterium_hallii_group, Marvinbryantia*. The proportion of C20:5n3 was negatively correlated with *Phoenicibacter*, *Lachnospiraceae_XPB1014_group*, *Christensenellaceae_R-7_group*, *norank_f__Lachnospiraceae*, but was positively correlated with *Prevotella*, *Marvinbryantia*, *Eubacterium_hallii_group*, and *Phascolarctobacterium*.

**Figure 7:**
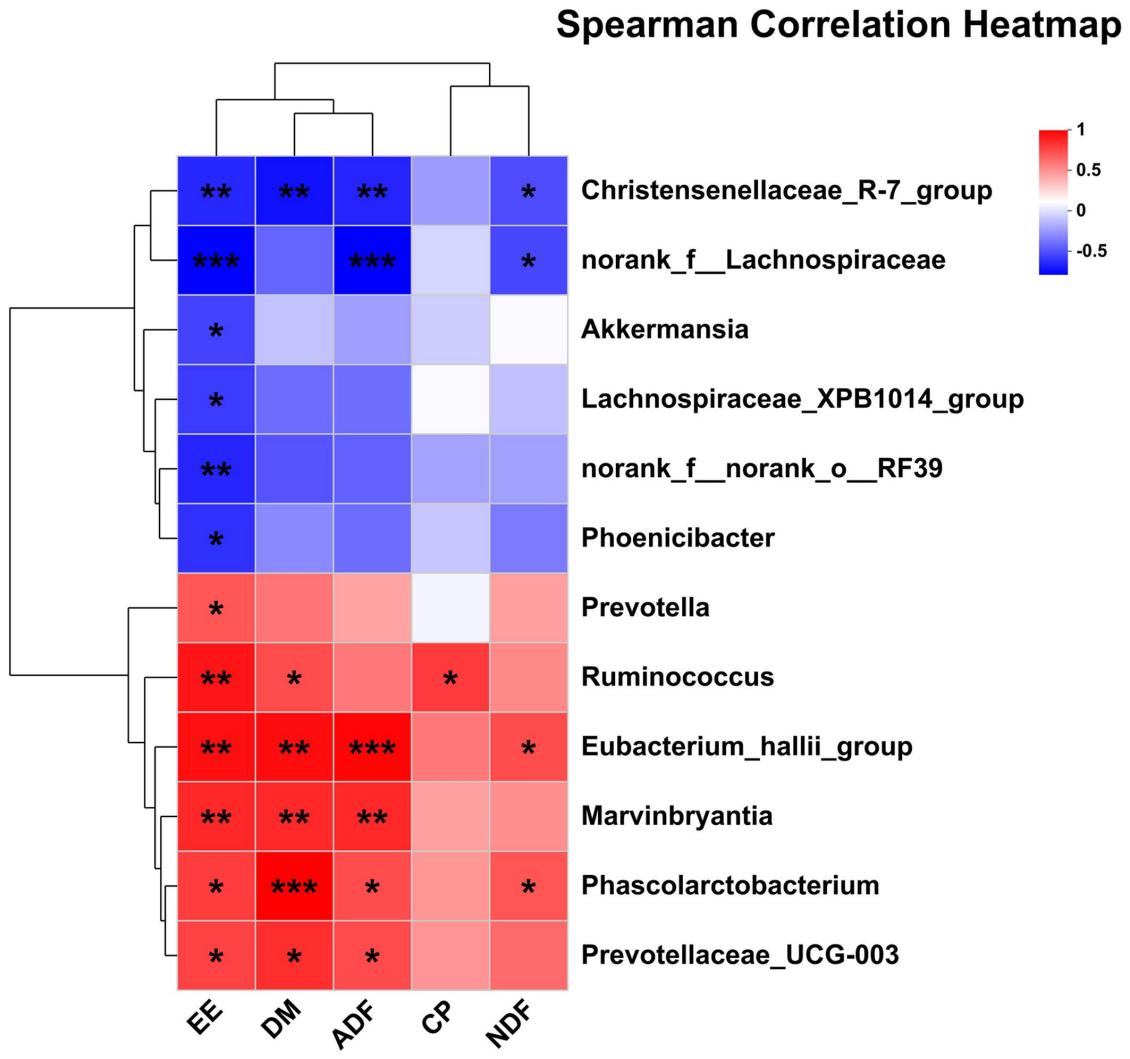
Pearson correlation analysis of differential bacteria genus with nutrient digestibility. Red indicates a positive correlation; the blue indicates a negative correlation. ***p ≤ 0.001, **0.001 < *p* ≤ 0.01, *0.01 < *p* ≤ 0.05.

The correlations between the significantly differential rectal bacteria and nutrient digestibility are presented in Figure 7. EE was negatively associated with *Christensenellaceae_R-7_group*, *norank_f__Lachnospiraceae*, *Lachnospiraceae_XPB1014_group*, *norank_f__norank_o__RF39*, *Phoenicibacter*, *Akkermansia* but was positively associated with *Ruminococcus*, *Marvinbryantia*, *Eubacterium_hallii_group*, *Phascolarctobacterium*, *Prevotellaceae_UCG-003*, and *Prevotella*. DM was also negatively associated with *Christensenellaceae_R-7_group* and positively associated with *Ruminococcus*, *Marvinbryantia*, *Eubacterium_hallii_group*, *Phascolarctobacterium*, and *Prevotellaceae_UCG-003*. ADF was negatively associated with *Christensenellaceae_R-7* and *norank_f__Lachnospiraceae*, but positively associated with *Marvinbryantia*, *Eubacterium_hallii_group*, *Phascolarctobacterium*, and *Prevotellaceae_UCG-003*. NDF was also negatively associated with *Christensenellaceae_R-7* and *norank_f__Lachnospiraceae*, but positively associated with *Eubacterium_hallii_group* and *Phascolarctobacterium*. CP was positively associated with *Ruminococcus*.

**Figure 8:**
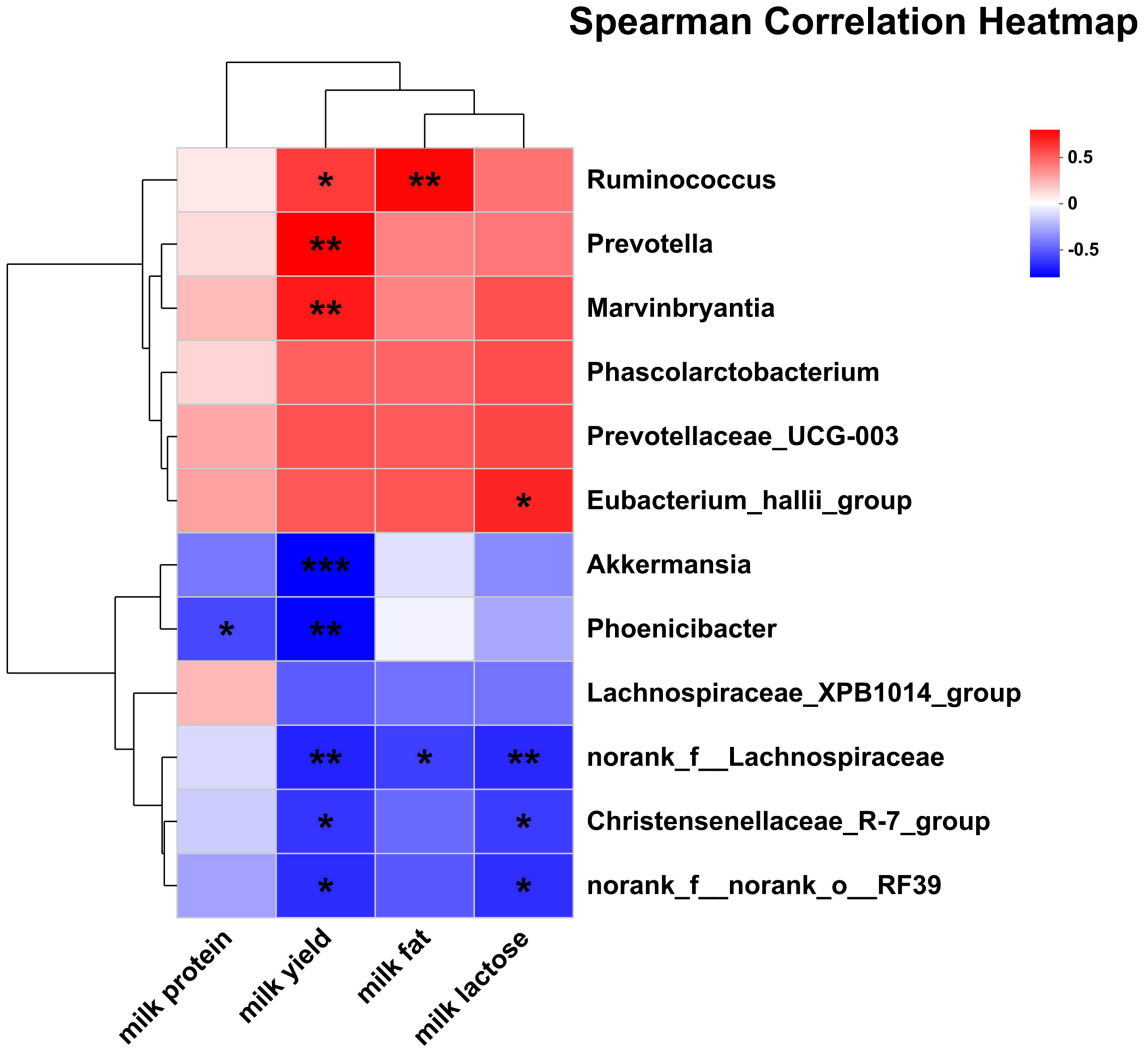
Pearson correlation analysis of differential bacteria genus with lactation performance. Red indicates a positive correlation; the blue indicates a negative correlation. ***p ≤ 0.001, **0.001 < *p* ≤ 0.01, *0.01 < *p* ≤ 0.05.

The correlations between the significantly differential rectal bacteria and lactation performance are presented in Figure 8. Milk yield was negatively associated with *Phoenicibacter*, *Akkermansia, Christensenellaceae_R-7_grou*p, *norank_f__Lachnospiraceae*, *norank_f__norank_o__RF39* but was positively associated with *Ruminococcus*, *Marvinbryantia*, and *Prevotella*. Milk protein was negatively associated with *Phoenicibacter*. Milk fat was negatively associated with *norank_f__Lachnospiraceae* but was positively associated with *Ruminococcus*. Milk lactose was negatively associated with *Christensenellaceae_R-7_grou*p, *norank_f__Lachnospiraceae*, *norank_f__norank_o__RF39* but was positively associated with *Eubacterium_hallii_group*.

## 4 Discussion

Donkey milk is characterized by low fat content yet high levels of essential fatty acids, with significantly higher PUFA concentrations compared to bovine or caprine milk (Wang et al., 2022). In our study, AOCP supplementation significantly elevated the levels of C18:1c9, C18:2c6, C20:5n3, MUFA, PUFA, and DFA beneficial to human health in donkey milk, thereby optimizing the FA profile. A similar trend in FA composition was observed in blood. However, the specific mechanisms underlying AOCP-mediated modulation of the FA profile in donkey milk remain unclear. Studies have demonstrated that *norank_f__Lachnospiraceae* participates in biohydrogenation (BH) processes (Zou et al., 2025). In the present study, *norank_f_Lachnospiraceae* was significantly enriched in the CON group relative to AOCP group, suggesting that AOCP may exert a suppressive effect on this BH-related bacterial genus. Spearman correlation analysis revealed that C20:5n3, DFA, and PUFA were negatively correlated with *norank_f_Lachnospiraceae*. These findings suggest that the elevated levels of C20:5n3, DFA, and PUFA following AOCP supplementation may be partially attributed to the observed inhibition of BH-related bacterial genera. However, as this correlative relationship was not directly verified in the present study, further research is essential to establish a definitive causal link. C16:0 in the mammary gland acts as a key limiting factor for the enhancement of milk fat yield (Benoit et al., 2024). The FAs involved in the synthesis of milk fat originate from two primary sources: *de novo* synthesis and exogenous uptake by mammary epithelial cells. Medium- and short-chain FAs (<16 carbons) along with approximately 50% of the C16:0 are synthesized *de novo* by mammary epithelial cells using acetate and butyrate as a substrate (Urrutia et al., 2019; Qi et al., 2014), and they constitute nearly half of the total FA utilized in milk fat synthesis. In contrast, long-chain FAs (>16 carbons), as well as about 50% of the C16:0 present in milk fat, are directly absorbed from the bloodstream by mammary epithelial cells (Hu et al., 2024). The *Eubacterium hallii group* is a bacterial group exhibiting butyrate-producing, cellulose-degrading, and antimicrobial properties (Engels et al., 2016; Song et al., 2014). In the present study, AOCP supplementation increased the abundance of the *Eubacterium hallii group*, elevated the production of acetate and butyrate, and concurrently raised milk fat content and C16:0 levels. These results suggest that AOCP supplementation could enhance *de novo* synthesis of C16:0 and milk fat synthesis by boosting acetate and butyrate levels, potentially through increasing the abundance of *Eubacterium hallii group*. However, these functional connections remain to be verified.The observed increase in C18:1c9 and MUFA levels may be attributed to enhanced FA desaturation mediated by AOCP. This mechanism is further supported by the corresponding elevation in SCD activity, which catalyzes a key step in MUFA synthesis. P/S is an important indicator for assessing dietary nutritional value, and the recommended value of P/S is 0.45 (Bouzaida et al., 2021), and greater than 0.45 is better. For cardiovascular protection and prophylaxis against atherosclerosis, the value of AI in foods must be lower than 1.0 (Frunză et al., 2025). In the present study, AOCP supplementation enhanced the P/S ratio, elevated DFA levels, and concurrently reduced the AI. These findings indicate that AOCP supplementation may optimize the FA profile of milk and enhance the content of FAs beneficial to human health. However, to date, no studies have reported the impact of AOCP on donkey milk fat quality. The present study is limited by a relatively small sample size of experimental animals and a lack of functional validation for key differential bacterial genera, warranting further investigation and exploration.

Current research on the effects of dietary AOCP supplementation on lactation performance in lactating donkeys remains limited. This study demonstrated that 0.5 g/kg DM AOCP supplementation significantly increased MY and elevated the production of milk fat, protein, lactose, TS, and SNF. The improvements may be partially attributed to enhanced nutrient digestion and metabolism, including increased digestibility of DM, EE, NDF, and ADF, as well as improved energy metabolic rate and DMI in lactating donkeys. As indicated previously, the underlying mechanism likely involves the increased abundance of the *Eubacterium hallii group* and elevated SCFA levels. Furthermore, the enhanced milk production performance is also linked to the increased abundance of other SCFA-producing bacteria. For instance, *Prevotella* efficiently degrades dietary fiber, primarily yielding acetate and succinate (Zhang et al., 2023). Symbiotic bacteria then convert succinate into propionate, which serves as a substrate for gluconeogenesis. This pathway generates glucose required for lactose synthesis. Additionally, *Ruminococcus* degrades fibrous substances, such as resistant starch, producing acetate and butyrate while enhancing intestinal barrier function (Crost et al., 2018). Consequently, the proliferation of these SCFA-producing bacteria increases rectal SCFA concentrations, elevates milk fat and lactose yields, and ultimately enhances milk production in donkeys through improved nutrient utilization. In our study, *Prevotella* showed positive correlations with EE digestibility and milk yield. *Ruminococcus* was positively correlated with the digestibility of DM, EE, and CP, as well as milk yield and milk fat content. These associations suggest the potential roles of these genera in enhancing nutrient utilization. In contrast, AOCP supplementation significantly reduced the relative abundance of opportunistic pathogens. Specifically, the relative abundance of *Streptococcus* and *norank_f_Lachnospiraceae* was significantly inhibited. *Streptococcus* is a Gram-positive genus known to induce systemic diseases such as meningitis and septicemia in monogastric animals (Lun et al., 2007). *Norank_f_Lachnospiraceae* has been reported for its positive correlation with uremic toxins and inflammatory markers (Hong et al., 2023). By suppressing these bacteria, AOCP may reduce energy expenditure on immune-metabolic processes, thereby potentially redirecting metabolic resources toward lactation. However, the specific mechanisms underlying this metabolic shift, including possible modulation of inflammatory pathways, require further comprehensive investigation.

Interestingly, although AOCP supplementation significantly increased the yields of milk protein, no significant increase in milk protein content was observed compared to the CON group. This indicates that the improvement in milk protein yield is mainly due to the overall increase in MY, rather than to a specific elevation in the protein content. The lack of change in protein content, together with the absence of improvement in milk protein synthesis efficiency, suggests that AOCP supplementation may not directly enhance mammary protein synthesis or improve the utilization of amino acids for protein production. Instead, the benefits appear to be more closely linked to enhancements in energy metabolism and nutrient partitioning directed toward general milk production and fat synthesis. This differential response underscores the specificity of the metabolic pathways modulated by AOCP. However, at present, there are few relevant studies, and the exact reasons need to be further explored in the future.

In summary, AOCP supplementation improved milk performance in lactating donkeys, which may be partially attributed to the optimization of intestinal bacterial structure. Such optimization is featured by an increased relative abundance of beneficial bacteria (e.g., *Eubacterium hallii group, Ruminococcus*, and *Prevotella*), as well as a reduced relative abundance of potentially pathogenic bacteria (e.g., *Streptococcus, norank_f__Lachnospiraceae*). However, that these inferences are drawn primarily from correlation analyses and have not been functionally validated. Therefore, further studies with larger sample sizes are necessary to corroborate these findings.

## 5 Conclusion

In conclusion, supplementation with 0.5 g/kg DM AOCP represents a novel nutritional strategy that may enhance milk production and milk fat yield, while also increasing the levels of C18:1c9, C18:2c6, and C20:5n3, as well as the U/S and P/S ratios in donkey milk. These improvements appear to be primarily mediated through enhanced digestion and metabolism of nutrients, modulation of enzymes related to lipid metabolism, and increased relative abundance of SCFA-producing bacteria. The optimized AOCP dosage established in this study provides a sustainable and eco-friendly feed additive for donkey farming, with the potential to enhance the economic viability of the dairy donkey industry. However, due to the limitation of a small sample size, future studies are needed to elucidate the exact mechanism by which AOCP regulates milk FA composition, with a focus on omics approaches such as lipid metabolomics and milk proteomics.

## Data Availability

The data presented in the study, specifically the raw 16S rRNA gene sequencing data, are deposited in the National Center for Biotechnology Information (NCBI) database, accession number PRJNA1332316.

## Footnotes

^1^ http://drive5.com/uparse/

^2^ http://www.mothur.org/wiki/Calculators
