# Supplementary material for: Optimization of Artemisia ordosica crude polysaccharides on milk fatty acid composition in lactating donkeys and their effects on rectal microbiome and lactation performance
Source: Front Microbiol. 2025 Nov 4;16:1682805. doi: 10.3389/fmicb.2025.1682805 (PMC12623351; doi:10.3389/fmicb.2025.1682805)
Supplement: Supplementary file 1 [file Table_1.DOCX]

**Supplementary Material**

**Table S1.** Effects of AOCP on fatty acid composition in blood of lactating donkeys (%，TFA)

| Items^3^ | CON^1^ | AOCP^1^ | SEM^2^ | *P*-value |
| --- | --- | --- | --- | --- |
| SFA |  |  |  |  |
| C4:0 | 0.45 | 0.52 | 0.064 | 0.459 |
| C6:0 | 0.23 | 0.22 | 0.019 | 0.543 |
| C8:0 | 1.09 | 0.98 | 0.063 | 0.274 |
| C10:0 | 0.05 | 0.04 | 0.007 | 0.244 |
| C11:0 | 0.13 | 0.10 | 0.028 | 0.390 |
| C12:0 | 0.03 | 0.04 | 0.010 | 0.886 |
| C13:0 | 0.03 | 0.04 | 0.003 | 0.133 |
| C14:0 | 0.47 | 0.50 | 0.057 | 0.706 |
| C15:0 | 0.17 | 0.17 | 0.011 | 0.909 |
| C16:0 | 15.34 | 16.31 | 0.497 | 0.198 |
| C17:0 | 0.40 | 0.37 | 0.010 | 0.138 |
| C18:0 | 19.23 | 18.68 | 0.286 | 0.197 |
| C20:0 | 0.43 | 0.44 | 0.010 | 0.528 |
| C21:0 | 0.06^a^ | 0.04^b^ | 0.004 | 0.003 |
| C22:0 | 0.25^a^ | 0.21^b^ | 0.009 | 0.021 |
| C23:0 | 0.52 | 0.55 | 0.016 | 0.303 |
| C24:0 | 0.36^a^ | 0.31^b^ | 0.012 | 0.023 |
| MUFA |  |  |  |  |
| C14:1 | 0.13 | 0.13 | 0.033 | 0.886 |
| C15:1 | 0.04 | 0.04 | 0.007 | 0.598 |
| C16:1 | 0.66^b^ | 0.77^a^ | 0.021 | 0.003 |
| C17:1 | 0.30^a^ | 0.24^b^ | 0.011 | 0.004 |
| C18:1t9 | 0.08 | 0.08 | 0.008 | 0.723 |
| C18:1c9 | 10.42^b^ | 11.49^a^ | 0.215 | 0.006 |
| C20:1 | 0.36 | 0.35 | 0.052 | 0.266 |
| C22:1 | 4.75^a^ | 3.77^b^ | 0.112 | <0.001 |
| C24:1 | 0.42 | 0.45 | 0.040 | 0.386 |
| n-6 PUFA |  |  |  |  |
| C18:2t6 | 0.04 | 0.03 | 0.004 | 0.175 |
| C18:2c6 | 38.14^b^ | 41.49^a^ | 0.899 | 0.025 |
| C18:3n6 | 0.01 | 0.01 | 0.001 | 0.110 |
| C20:2n6 | 0.34a | 0.31b | 0.007 | 0.042 |
| C20:3n6 | 0.13^b^ | 0.15^a^ | 0.002 | 0.003 |
| C20:4n6 | 0.07 | 0.06 | 0.010 | 0.654 |
| C22:2n6 | 0.16a | 0.13b | 0.004 | <0.001 |
| n-3 PUFA |  |  |  |  |
| C18:3n3 | 0.60 | 0.63 | 0.016 | 0.253 |
| C20:3n3 | 0.17^a^ | 0.13^b^ | 0.005 | <0.001 |
| C20:5n3 | 0.07^b^ | 0.10^a^ | 0.007 | 0.007 |
| C22:6n3 | 0.13 | 0.13 | 0.010 | 0.465 |
| Sum and Ratio |  |  |  |  |
| SFA | 42.98^a^ | 39.52^b^ | 0.895 | 0.024 |
| UFA | 57.02^b^ | 60.49^a^ | 0.895 | 0.024 |
| MUFA | 17.16 | 17.31 | 0.220 | 0.665 |
| PUFA | 39.86^b^ | 43.18^a^ | 0.895 | 0.026 |
| n-3 PUFA | 0.98^b^ | 1.02^a^ | 0.008 | 0.028 |
| n-6 PUFA | 38.89^b^ | 42.19^a^ | 0.897 | 0.026 |
| n-3 LCPUFA | 0.37 | 0.36 | 0.009 | 0.594 |
| n-6 LCPUFA | 0.70 | 0.65 | 0.013 | 0.052 |
| n-6/n-3 | 39.59 | 40.49 | 0.720 | 0.428 |
| U/S | 1.33^b^ | 1.54^a^ | 0.054 | 0.026 |
| P/S | 0.93^b^ | 1.10^a^ | 0.044 | 0.026 |
| Desirable fatty acid (DFA) | 76.25 | 79.16 | 1.040 | 0.086 |
| Atherogenicity Index (AI) | 0.30 | 0.30 | 0.014 | 0.988 |
| Thrombogenic Index (TI) | 1.13 | 1.08 | 0.027 | 0.220 |
| (C18:0 + C18:1)/C16:0 | 1.95 | 1.86 | 0.053 | 0.292 |

^a,b^Means within a row with different letters are significantly different (*P* < 0.05).

^1^CON = control (basal diet); AOCP = *Artemisia ordosica* crude polysaccharides (basal diet with 0.5 g/kg DM AOCP).

^2^SEM = standard error of the mean.

^3^SFA = saturated fatty acid; UFA = unsaturated fatty acid; MUFA = monounsaturated fatty acid; PUFA = polyunsaturated fatty acids.

**Table S2.** Effects of AOCP on fatty acid composition and yield in milk of lactating donkeys

| Items^3^ | milk FA composition (%，TFA) | | | |  | milk FA yield (g/day) | | | |
| --- | --- | --- | --- | --- | --- | --- | --- | --- | --- |
|  | CON^1^ | AOCP^1^ | SEM2 | *P*-value |  | CON^1^ | AOCP^1^ | SEM2 | *P*-value |
| SFA |  |  |  |  |  |  |  |  |  |
| C4:0 | 0.19 | 0.18 | 0.019 | 0.658 |  | 0.70 | 0.69 | 0.073 | 0.938 |
| C6:0 | 0.28 | 0.30 | 0.006 | 0.086 |  | 1.05 | 0.17 | 0.061 | 0.193 |
| C8:0 | 5.05 | 5.06 | 0.214 | 0.960 |  | 18.81 | 19.96 | 1.545 | 0.607 |
| C10:0 | 12.30 | 11.62 | 0.651 | 0.499 |  | 45.67 | 45.90 | 3.826 | 0.967 |
| C11:0 | 0.03a | 0.01b | 0.002 | 0.003 |  | 0.11^a^ | 0.05^b^ | 0.005 | 0.002 |
| C12:0 | 9.89 | 9.15 | 0.289 | 0.156 |  | 36.57 | 35.89 | 2.096 | 0.820 |
| C13:0 | 0.09 | 0.15 | 0.025 | 0.224 |  | 0.31 | 0.57 | 0.101 | 0.103 |
| C14:0 | 6.69a | 6.06b | 0.073 | <0.001 |  | 24.76 | 23.66 | 0.984 | 0.443 |
| C15:0 | 0.30 | 0.34 | 0.018 | 0.118 |  | 1.12 | 1.34 | 0.094 | 0.114 |
| C16:0 | 18.51^b^ | 20.14^a^ | 0.454 | 0.026 |  | 68.39^b^ | 78.69^a^ | 2.994 | 0.039 |
| C17:0 | 0.27 | 0.27 | 0.006 | 0.719 |  | 0.99 | 1.06 | 0.057 | 0.370 |
| C18:0 | 1.61 | 1.63 | 0.042 | 0.782 |  | 5.97 | 6.40 | 0.357 | 0.409 |
| C20:0 | 0.03 | 0.04 | 0.001 | 0.097 |  | 0.13 | 0.15 | 0.008 | 0.124 |
| C21:0 | 0.01 | 0.01 | 0.004 | 0.476 |  | 0.05 | 0.03 | 0.018 | 0.767 |
| C22:0 | 0.01 | 0.01 | 0.001 | 0.669 |  | 0.05 | 0.05 | 0.003 | 0.930 |
| C23:0 | 0.08 | 0.08 | 0.001 | 1.000 |  | 0.30 | 0.32 | 0.015 | 0.407 |
| C24:0 | 0.00 | 0.00 | 0.001 | 0.783 |  | 0.02 | 0.02 | 0.005 | 0.370 |
| MUFA |  |  |  |  |  |  |  |  |  |
| C14:1 | 0.23 | 0.23 | 0.012 | 0.724 |  | 0.86 | 0.87 | 0.045 | 0.876 |
| C15:1 | 0.01 | 0.01 | 0.001 | 1.000 |  | 0.02 | 0.02 | 0.006 | 0.977 |
| C16:1 | 2.17b | 2.67a | 0.068 | 0.001 |  | 8.06^b^ | 10.43^a^ | 0.473 | 0.005 |
| C17:1 | 0.01 | 0.01 | 0.001 | 0.072 |  | 0.02 | 0.03 | 0.005 | 0.139 |
| C18:1t9 | 0.05b | 0.06a | 0.002 | 0.008 |  | 0.17^b^ | 0.22^a^ | 0.012 | 0.037 |
| C18:1c9 | 18.05b | 20.02a | 0.597 | 0.043 |  | 66.90^b^ | 78.02^a^ | 3.369 | 0.042 |
| C20:1 | 0.21b | 0.25a | 0.006 | 0.009 |  | 0.79^b^ | 0.96^a^ | 0.039 | 0.011 |
| C22:1 | 0.09a | 0.08b | 0.003 | 0.010 |  | 0.33 | 0.29 | 0.017 | 0.166 |
| C24:1 | 0.01 | 0.01 | 0.001 | 0.377 |  | 0.04 | 0.05 | 0.004 | 0.194 |
| n-6 PUFA |  |  |  |  |  |  |  |  |  |
| C18:2t6 | 0.01 | 0.01 | 0.001 | 0.110 |  | 0.03^b^ | 0.04^a^ | 0.003 | 0.059 |
| C18:2c6 | 17.41b | 18.84a | 0.442 | 0.043 |  | 64.45 | 73.95 | 3.530 | 0.087 |
| C18:3n6 | 0.02 | 0.02 | 0.003 | 0.508 |  | 0.07 | 0.06 | 0.014 | 0.800 |
| C20:2n6 | 0.43a | 0.40b | 0.005 | 0.021 |  | 1.57 | 1.57 | 0.078 | 0.966 |
| C20:3n6 | 0.04 | 0.04 | 0.001 | 0.102 |  | 0.13^b^ | 0.16^a^ | 0.008 | 0.019 |
| C20:4n6 | 0.01 | 0.01 | 0.001 | 0.702 |  | 0.03 | 0.03 | 0.005 | 0.876 |
| C22:2n6 | 0.03 | 0.03 | 0.001 | 0.113 |  | 0.13 | 0.11 | 0.008 | 0.323 |
| n-3 PUFA |  |  |  |  |  |  |  |  |  |
| C18:3n3 | 3.33 | 3.37 | 0.128 | 0.843 |  | 12.34 | 13.20 | 0.728 | 0.427 |
| C20:3n3 | 0.10a | 0.09b | 0.001 | 0.001 |  | 0.36 | 0.34 | 0.017 | 0.407 |
| C20:5n3 | 0.01b | 0.02a | 0.001 | <0.001 |  | 0.02^b^ | 0.06^a^ | 0.002 | <0.001 |
| C22:6n3 | 0.02 | 0.03 | 0.004 | 0.184 |  | 0.07 | 0.10 | 0.019 | 0.225 |
| Sum and Ratio |  |  |  |  |  |  |  |  |  |
| SFA | 57.78a | 53.84b | 0.701 | 0.002 |  | 213.91 | 211.05 | 9.792 | 0.841 |
| UFA | 42.22b | 46.16a | 0.701 | 0.002 |  | 156.39^b^ | 180.50^a^ | 7.340 | 0.040 |
| MUFA | 20.82b | 23.32a | 0.618 | 0.018 |  | 77.18^b^ | 90.89^a^ | 3.755 | 0.027 |
| PUFA | 21.40^b^ | 22.84^a^ | 0.468 | 0.024 |  | 79.20 | 89.61 | 4.263 | 0.114 |
| n-3 PUFA | 3.46 | 3.50 | 0.125 | 0.820 |  | 12.80 | 13.69 | 0.734 | 0.408 |
| n-6 PUFA | 17.94b | 19.34a | 0.442 | 0.047 |  | 66.40 | 75.91 | 3.610 | 0.093 |
| n-3 LCPUFA | 0.12 | 0.13 | 0.005 | 0.498 |  | 0.45 | 0.50 | 0.026 | 0.282 |
| n-6 LCPUFA | 0.50a | 0.48^b^ | 0.006 | 0.036 |  | 1.86 | 1.87 | 0.089 | 0.930 |
| n-6/n-3 | 5.24 | 5.53 | 0.165 | 0.281 |  | 5.24 | 5.53 | 0.165 | 0.282 |
| U/S | 0.74b | 0.85a | 0.025 | 0.009 |  | 0.73^b^ | 0.86^a^ | 0.024 | 0.011 |
| P/S | 0.38b | 0.42a | 0.113 | 0.015 |  | 0.37^b^ | 0.43^a^ | 0.014 | 0.042 |
| Desirable fatty acid (DFA) | 43.83^b^ | 47.79^a^ | 0.684 | 0.007 |  | 162.35^b^ | 186.90^a^ | 7.609 | 0.043 |
| Atherogenicity Index (AI) | 1.31^a^ | 1.16^b^ | 0.030 | 0.007 |  | 1.31^a^ | 1.16^b^ | 0.030 | 0.007 |
| Thrombogenic Index (TI) | 0.90 | 0.87 | 0.027 | 0.544 |  | 0.90 | 0.88 | 0.027 | 0.502 |
| (C18:0 + C18:1)/C16:0 | 1.07 | 1.08 | 0.030 | 0.775 |  | 1.07 | 1.08 | 0.030 | 0.775 |

^a,b^Means within a row with different letters are significantly different (*P* < 0.05).

^1^CON = control (basal diet); AOCP = *Artemisia ordosica* crude polysaccharides (basal diet with 0.5 g/kg DM AOCP).

^2^SEM = standard error of the mean.

^3^SFA = saturated fatty acid; UFA = unsaturated fatty acid; MUFA = monounsaturated fatty acid; PUFA = polyunsaturated fatty acids.
